# Learning From Biological and Computational Machines: Importance of SARS-CoV-2 Genomic Surveillance, Mutations and Risk Stratification

**DOI:** 10.3389/fcimb.2021.783961

**Published:** 2021-12-24

**Authors:** Shikha Bhat, Anuradha Pandey, Akshay Kanakan, Ranjeet Maurya, Janani Srinivasa Vasudevan, Priti Devi, Partha Chattopadhyay, Shimpa Sharma, Rajesh J. Khyalappa, Meghnad G. Joshi, Rajesh Pandey

**Affiliations:** ^1^ INtegrative GENomics of HOst-PathogEn (INGEN-HOPE) Laboratory, CSIR-Institute of Genomics and Integrative Biology (CSIR-IGIB), New Delhi, India; ^2^ Birla Institute of Technology and Science, Pilani, India; ^3^ Academy of Scientific and Innovative Research (AcSIR), Ghaziabad, India; ^4^ D. Y. Patil Medical College Kolhapur, Kasaba Bawada, Kolhapur, India

**Keywords:** COVID-19, SARS-CoV-2, genomic surveillance, risk stratification, machine learning, healthcare

## Abstract

The global coronavirus disease 2019 (COVID-19) pandemic has demonstrated the range of disease severity and pathogen genomic diversity emanating from a singular virus (severe acute respiratory syndrome coronavirus 2, SARS-CoV-2). This diversity in disease manifestations and genomic mutations has challenged healthcare management and resource allocation during the pandemic, especially for countries such as India with a bigger population base. Here, we undertake a combinatorial approach toward scrutinizing the diagnostic and genomic diversity to extract meaningful information from the chaos of COVID-19 in the Indian context. Using methods of statistical correlation, machine learning (ML), and genomic sequencing on a clinically comprehensive patient dataset with corresponding with/without respiratory support samples, we highlight specific significant diagnostic parameters and ML models for assessing the risk of developing severe COVID-19. This information is further contextualized in the backdrop of SARS-CoV-2 genomic features in the cohort for pathogen genomic evolution monitoring. Analysis of the patient demographic features and symptoms revealed that age, breathlessness, and cough were significantly associated with severe disease; at the same time, we found no severe patient reporting absence of physical symptoms. Observing the trends in biochemical/biophysical diagnostic parameters, we noted that the respiratory rate, total leukocyte count (TLC), blood urea levels, and C-reactive protein (CRP) levels were directly correlated with the probability of developing severe disease. Out of five different ML algorithms tested to predict patient severity, the multi-layer perceptron-based model performed the best, with a receiver operating characteristic (ROC) score of 0.96 and an F1 score of 0.791. The SARS-CoV-2 genomic analysis highlighted a set of mutations with global frequency flips and future inculcation into variants of concern (VOCs) and variants of interest (VOIs), which can be further monitored and annotated for functional significance. In summary, our findings highlight the importance of SARS-CoV-2 genomic surveillance and statistical analysis of clinical data to develop a risk assessment ML model.

## Introduction

Since December 2019, a novel coronavirus, severe acute respiratory syndrome coronavirus 2 (SARS-CoV-2), has been observed to cause coronavirus disease 2019 (COVID-19). Thereafter, we have observed 254 million COVID-19 cases, 5.12 million deaths, 4 SARS-CoV-2 variants of concern (VOCs), 5 variants of interest (VOIs), and 11 other variants under monitoring. In India, we have witnessed 0.465 million deaths due to COVID-19 until mid-November 2021 (WHO Coronavirus (COVID-19) Dashboard). During the second surge of COVID-19 by the 21A (Delta) variant (Dhar et al., 2021; Mlcochova et al., 2021), India witnessed the effects of an overburdened healthcare infrastructure. Similarly, in many parts of the world, the COVID-19 pandemic has caused distress and resulted in mortalities that are not only direct consequences of the disease but also as associated consequences of an overburdened medical infrastructure (Xie et al., 2020; Usher, 2021; Singh, 2021).

Patient severity of COVID-19 ranges from being asymptomatic to symptomatic and a fraction resulting in mortality, with nearly 1.3% patients succumbing to the disease in India (Johns Hopkins Coronavirus Resource Center). Thereby, a majority of the patients exhibited varying levels of intermediate severity. These patients reported symptoms ranging from cough, fever, breathlessness to chest pain and loss of movement (Huang et al., 2020). Effective triage of these patients reaching a healthcare facility at the early phases of the disease when the symptoms are mild is crucial for effective healthcare management. This can help in improved resource allocation such as hospital beds, respiratory support, and targeted drugs. Furthermore, it can also help in administering drugs that are effective only at a particular severity stage (Beigel et al., 2020).

The novel coronavirus, similar to other coronaviruses, has been observed primarily to be spreading *via* fomites and direct human-to-human interactions. Transmission through the fecal-oral route and intrauterine vertical transfer have also been reported for SARS-CoV-2, as the pathogen has been demonstrated to stabilize in human digestive tract. Fecal matter and wastewater have been shown to contain active viral particles, which are shed even after the upper respiratory tract turns negative for viral RNA (Rana et al., 2021). This has led to initiatives of wastewater surveillance for SARS-CoV-2 to complement the naso/oropharyngeal sampling-based genomic surveillance. Similar to other RNA viruses, SARS-CoV-2 exists in the global population as a group of similar strains due to its rate of mutation acquisition. This ability to acquire mutations and thereby gain clinically and epidemiologically significant functions is a global concern during this pandemic (Barr and Fearns, 2016; Kanakan et al., 2020). To track this evolution of SARS-CoV-2 and flag the emergence of novel mutations, many countries have initiated integrative SARS-CoV-2 genome surveillance initiatives. This includes the COVID-19 Genomics Consortium in the UK (COVID-19 Genomics UK Consortium), SARS-CoV-2 Sequencing for Public Health Emergency Response, Epidemiology, and Surveillance (SPHERES) in the United States (SPHERES|CDC), and the Indian SARS-CoV-2 Genomics Consortium (INSACOG) (INSACOG|Department of Biotechnology). Such combined efforts of sequencing have facilitated SARS-CoV-2 genome sequences from their respective regions for functional insights (GISAID - Initiative). This aids in the early detection of potential gain-of-function mutations and tracking the selection of these mutations in emerging SARS-CoV-2 strains. Based on subsequent functional annotations of these mutations through the combination of *in silico* and *in vitro* experiments, SARS-CoV-2 strains have been classified according to the risks they pose to global health as VOCs and VOIs (Oude Munnink et al., 2021).

Pathological findings of COVID-19 have been implicated in the prediction of their severity capacity in many different studies using various approaches. The statistical significance of many such factors has been reported in different population cohorts. Studies have highlighted the statistical significance of the pathological findings of COVID-19 (Hu et al., 2020; Li et al., 2020; Yang et al., 2020; Zhou et al., 2020), including Indian cohorts (Gupta et al., 2020; Khan M. et al., 2020). Similarly, machine learning (ML)-based models have been built for COVID-19 using cohorts from North America (Cheng et al., 2020a; Khan A. I. et al., 2020), South Korea (Kim et al., 2020), and China (Wu et al., 2020; Karthikeyan et al., 2021). Due to the overwhelming host genetic, immunological, environmental, and healthcare factors, population-level differences in COVID-19 manifestations have been observed (Sorci et al., 2020; Lo et al., 2021; Maurya et al., 2021; Ong et al., 2021; Zhang et al., 2021), therefore necessitating the development of a severity prediction algorithm based on an Indian cohort, which is presented in this manuscript. At the same time, it is important to integrate different aspects of COVID-19 rather than only one at a time.

Using our dataset, inclusive of a broad range of biochemical test reports over multiple demographic and clinical observations of COVID-19 patients, we present an approach to narrow down the diverse clinical factors of COVID-19 into a few functionally important variables. We analyzed the early-onset symptoms presented by COVID-19 patients associated with the development of severe disease and further used statistical methods to identify important biomarkers for severity progression of the disease. It is important to use and analyze clinical data as these can provide insightful information about the disease patterns, risk factors, and outcomes of treatment (Nass et al., 2009). We devised a ML pipeline to predict the outcomes of patients as severe or mild using the nested cross-validation (nested CV) algorithm (Bhargava et al., 2020; Hao et al., 2020; Liang et al., 2020; Gupta R. K. et al., 2021). We studied five computational learning models, namely, logistic regression, random forest, XGBoost, support vector machine (SVM), and multilayer perceptron. The predictive results of each model were compared and analyzed, and together, they can be implemented in clinical settings to predict COVID-19 severity in patients during the early stages of SARS-CoV-2 infection. Patient risk stratification and identification of the relative contributions of specific risk factors to overall risk are two of the widest applications of ML in healthcare (Wiens and Shenoy, 2018). ML/deep learning (DL) promote a data-driven approach to common yet important problems such as patient categorization and can greatly improve the management of patients in hospitals (Ching et al., 2018). They also have the potential to increase the efficiency and minimize the failure rates in drug discovery and development (Vamathevan et al., 2019).

Beyond contextualizing our study by providing the SARS-CoV-2 genomic constitution of our cohort, we further highlight the mutational diversity of SARS-CoV-2 in the background of its phylogenetic diversity in the cohort. Herein, we observe the evolutionary selection of the low-frequency mutations by comparing our genomic data to current global mutational spectra. Therefore, this study aimed to provide additional information and context to early-onset symptoms and clinical features for the improved risk prediction of patients diagnosed with COVID-19 using a combination of statistical and ML techniques. At the same time, this study highlights the importance of genomic surveillance in identifying SARS-CoV-2 genome mutations, which can undergo evolutionary selection in the future.

## Methodology

### Data Acquisition and Pre-processing

The data used in the study were collected from 257 confirmed COVID-19 patients from DY Patil group of hospitals in Pune, Maharashtra, India. Patients were admitted in the hospital between July and September 2020. A confirmed COVID-19 case was defined by a positive real-time polymerase chain reaction (RT-PCR) test for SARS-CoV-2 infection. The patient records were collected and anonymized at the data warehouse of CSIR-IGIB. The electronic hospital data used in the study included multiple demographics, vitals, and biochemical test reports pertaining to the COVID-19 patients. This comprehensive dataset encompasses vital signs and patient demographic data on oxygen saturation, respiratory rate, body mass index (BMI), age, gender, comorbidities, and respiratory support levels. Detailed blood test reports of the levels of C-reactive protein (CRP), interleukin 6 (IL-6), total leukocyte count (TLC), D-dimer, and lactate dehydrogenase, among others, were also included in the study, with a total of 31 different test parameters. The data were then curated to exclude patients with missing values of critical parameters. The patients were categorized as *severe* or *mild* based on their disease outcomes, i.e., discharged or deceased and ventilatory support requirement, as respiratory failure is a well-known indicator of COVID-19 severity. All patients requiring ventilatory support or deceased were considered to be severe; the rest were categorized as mild. This resulted in a curated list of 175 patients, which has been used in this study.

### RT-qPCR

To obtain SARS-CoV-2 viral RNA, upper or lower respiratory tract secretions were used for obtaining naso/oropharyngeal swabs, which were preserved in a viral transport media (VTM) solution; for patients with a productive cough, liquified sputum samples were obtained to extract RNA. RNA extraction was performed using a silica column-based RNA extraction kit for cell-free bodily fluids (QIAmp viral mini kit, cat. no 52906; Qiagen, Hilden, Germany). Of the VTM solution, 200 μl was processed for lysing and viral enrichment, in accordance with the protocol in the kit (QIAamp Viral RNA Mini Handbook). RNA was eluted in RNase-free water after washing with wash buffers. Quantitative real-time polymerase chain reaction (RT-qPCR) for SARS-CoV-2 detection was performed using the TRUPCR SARS-CoV-2 kit (cat. no 3B304; 3B BlackBio Biotech India Ltd., Bhopal, India). For RT-qPCR, 10 μl RNA was added to 15 μl of the reaction mixture in accordance with the kit protocol. The qPCR reaction was run on Rotor-Gene Q (Qiagen) using the recommended cycling conditions. To designate a patient as positive, a cycle threshold (*C*_t_) value of ≤35 was considered.

### SARS-CoV-2 Whole-Genome Sequencing

RNA was checked for the presence of sufficient quality and quantity for sequencing. A sequencing library was prepared from the RNA samples for sequencing on the Oxford Nanopore or Illumina-MiSeq platforms. Briefly, double-stranded cDNA was synthesized using 100 ng of total RNA. Herein, first-strand cDNA was synthesized using Superscript IV (cat. no. 18091050; Thermo Fisher Scientific, Waltham, MA, USA) and second-strand using DNA polymerase-I large (Klenow) fragment (cat. no. M0210S; New England Biolabs, Ipswich, MA, USA) after RNase H digestion of RNA in the first-strand. Purification of the double-stranded cDNA was carried out using AMPure XP beads (cat. no. A63881; Beckman Coulter, Brea, CA, USA).

Further sequencing library was prepared according to the Oxford Nanopore Technology (ONT) library preparation protocol *PCR tiling of COVID-19 virus* (version: PTC_9096_v109revE_06Feb2020) for Oxford Nanopore sequencing. Here, 100 ng of the purified cDNA, 200 ng of multiplexed PCR amplicons, and 200 ng of end-prepped samples were taken to perform ARTIC multiplex PCR, end repair, and barcode ligation, respectively. The final library was quantified on a Qubit High Sensitivity DNA kit (cat. no. Q32854) and sequenced using the MinION Mk1C platform.

For sequencing on the Illumina MiSeq platform, the sequencing library was prepared using the Illumina DNA Prep with Enrichment protocol (doc. no. 1000000048041v05; Illumina, San Diego, CA, USA). For this purpose, 100 ng of purified cDNA was used to prepare the library following tagmentation, indexing, enrichment, PCR amplification, and purification. Enrichment was performed using the Illumina Respiratory Virus Oligo Panel (cat no. 20042472; Illumina). The quality and the quantity of the sequencing library were checked using the Agilent 2100 Bioanalyzer with high sensitivity DNA chip (cat. no. 5067-4626; Agilent, Santa Clara, CA, USA) and the Qubit dsDNA High Sensitivity DNA kit, respectively. A loading concentration of 10 pM was prepared by denaturing and diluting the libraries in accordance with the MiSeq System Denature and Dilute Libraries Guide (document no. 15039740, v10; Illumina). Sequencing was performed on the MiSeq system using the MiSeq Reagent Kit v3 (150 cycles) at 2 × 75 bp read length.

### Sequencing Data Analysis

To analyze MinION raw fast5 files until variant calling, the ARTIC end-to-end pipeline was used. Base calling was performed using Guppy basecaller v3.2.4 (https://nanoporetech.com/nanopore-sequencing-data-analysis) with a quality cutoff Phred score of >7 on a GPU-Linux accelerated computing machine. Demultiplexed fastq reads were normalized by a read length of 300–500 bp for further downstream analysis and aligned to the SARS-CoV-2 reference (MN908947.3) using the aligner Minimap2 v2.17 (Li, 2018). To index the raw fast5 files for variant calling and to create consensus fasta, Nanopolish v0.13.3 (Loman et al., 2015), SAMtools v1.7, and BCFtools v1.8 (Danecek et al., 2021) were used over the normalized minimap2 output.

All fastq files generated from Illumina sequencing were checked for quality using FastQC v0.11.9. A threshold of Phred score >20 was used for filtering the reads from all samples. Subsequently, adapter trimming was performed using TrimGalore v0.6.6 and alignment of the sequences was performed using HISAT2 v2.2.1 (Kim et al., 2019) on human data build hg38 (Kim et al., 2015). SAMtools v1.12 was used to remove aligned sequences. Henceforth, only unaligned sequences were taken into consideration. BCFTools v1.12 was used to generate the consensus fasta and variant calling.

### Phylogenetic Analysis

The Wuhan reference genome for SARS-CoV-2 (NC_045512.2) was used to perform multiple sequence alignments of 92 SARS-CoV-2 genomes using MAFFT (v7.475) (Katoh et al., 2002). The alignment was manually trimmed and a phylogenetic tree was constructed using MEGA-X (Tamura et al., 2007). SARS-CoV-2 clades were assigned using Nextclade (https://clades.nextstrain.org/). The phylogenetic analysis was visualized with FIGTREE software (http://tree.bio.ed.ac.uk/software/figtree/).

### Mutation Analysis

The vcf file was used to obtain the top most frequent and least frequent mutations in the samples, and snpEff v5.0 (Cingolani et al., 2012) was utilized to perform variant annotation such as the variant definitions. The SnpEff database was created with “SnpEff build” using the Wuhan reference NC_045512.2. Furthermore, global frequency of the mutations was checked against a global dataset available at 2019 Novel Coronavirus Resource (2019nCoVR), CNCB (Song et al., 2020). Once the annotated VCF was generated, a lollipop plot representing the low-frequency (lower quartile) and-high frequency (upper quartile) mutations was generated in R v4.1.0 using the g3viz (Guo, 2021), rtracklayer (Michael Lawrence, 2017), and trackViewer (Ou and Zhu, 2019) packages, followed by data visualization using the ggplot2 package (ggplot2 version 3.3.5, 2021). Inkscape was utilized to modify the figures (Draw Freely|Inkscape). Variant position along the SARS-CoV-2 genome is indicated in the plot, which was used to compare the high- and low-frequency mutations of the cohort study with the global frequency.

### Statistical Analysis

Continuous and categorical variables are represented as median (interquartile range, IQR) and *n* (%). We applied the point-biserial correlation to compare continuous and dichotomous variables. Fisher’s exact test was used to comparing categorical variables of gender, breathlessness, cough, diabetes, fever, hypertension, heart conditions, and presence of other comorbidities. Using these tests, the *p*-values of all features were calculated. Multiple testing correction was done using the Bonferroni test with an alpha value of 0.05. Data pre-processing was done using pandas (https://pandas.pydata.org/pandas-docs/stable/) and NumPy (Bisong, 2019). Statistical analysis was conducted with SciPy (SciPy v1.7.1; https://docs.scipy.org/doc/scipy/reference/) and statsmodels (https://www.researchgate.net/publication/264891066_Statsmodels_Econometric_and_Statistical_Modeling_with_Python), and the findings are visually represented using the Matplotlib (Matplotlib 3.4.3; https://matplotlib.org/) and Seaborn (Waskom, 2021) libraries in Python (Python.org).

### Machine Learning Pipeline

**Figure 1:**
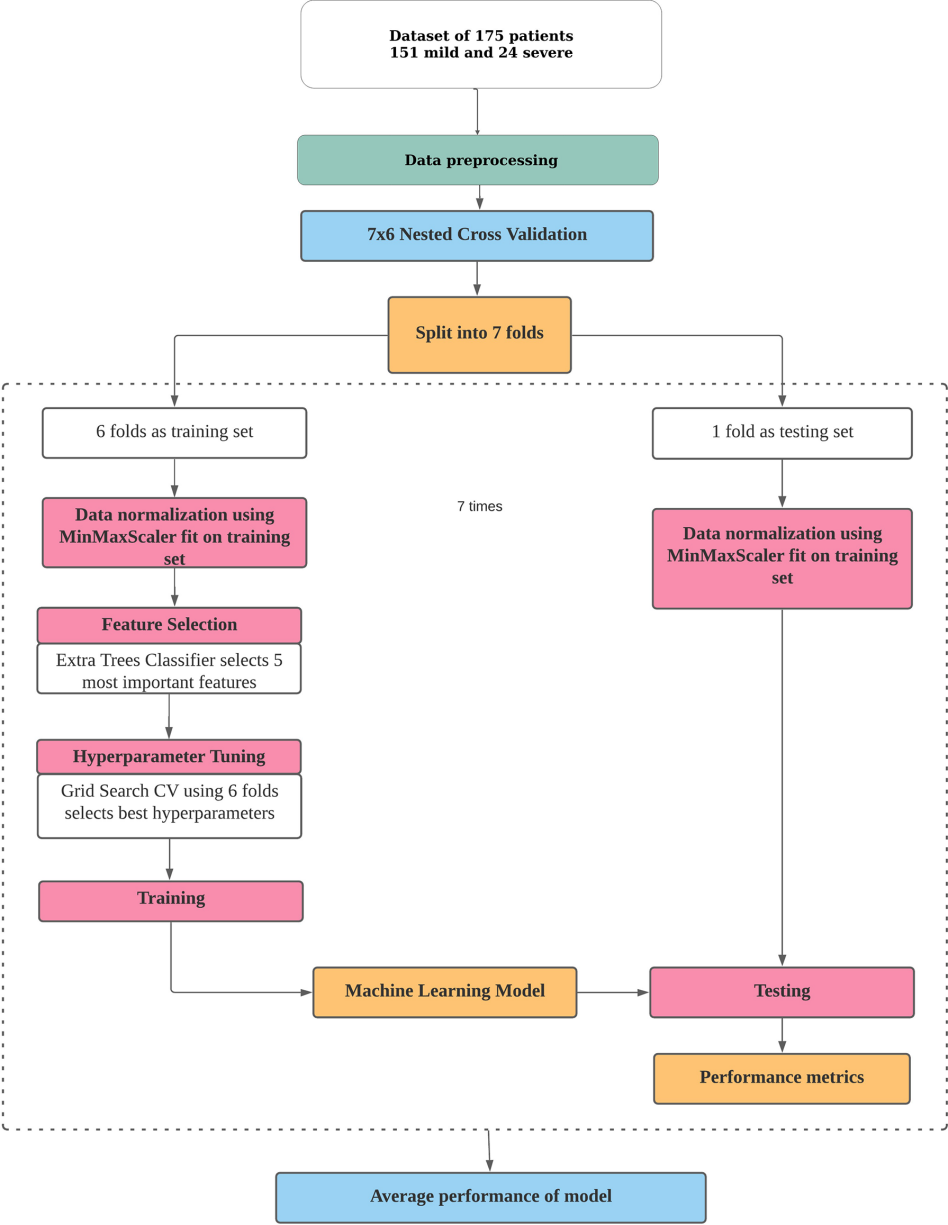
Pipeline for building a machine learning model for risk prediction. The processes of data pre-processing, data normalization, feature selection, hyperparameter tuning, and training and testing of a model in nested cross-validation are depicted.

**Figure 2:**
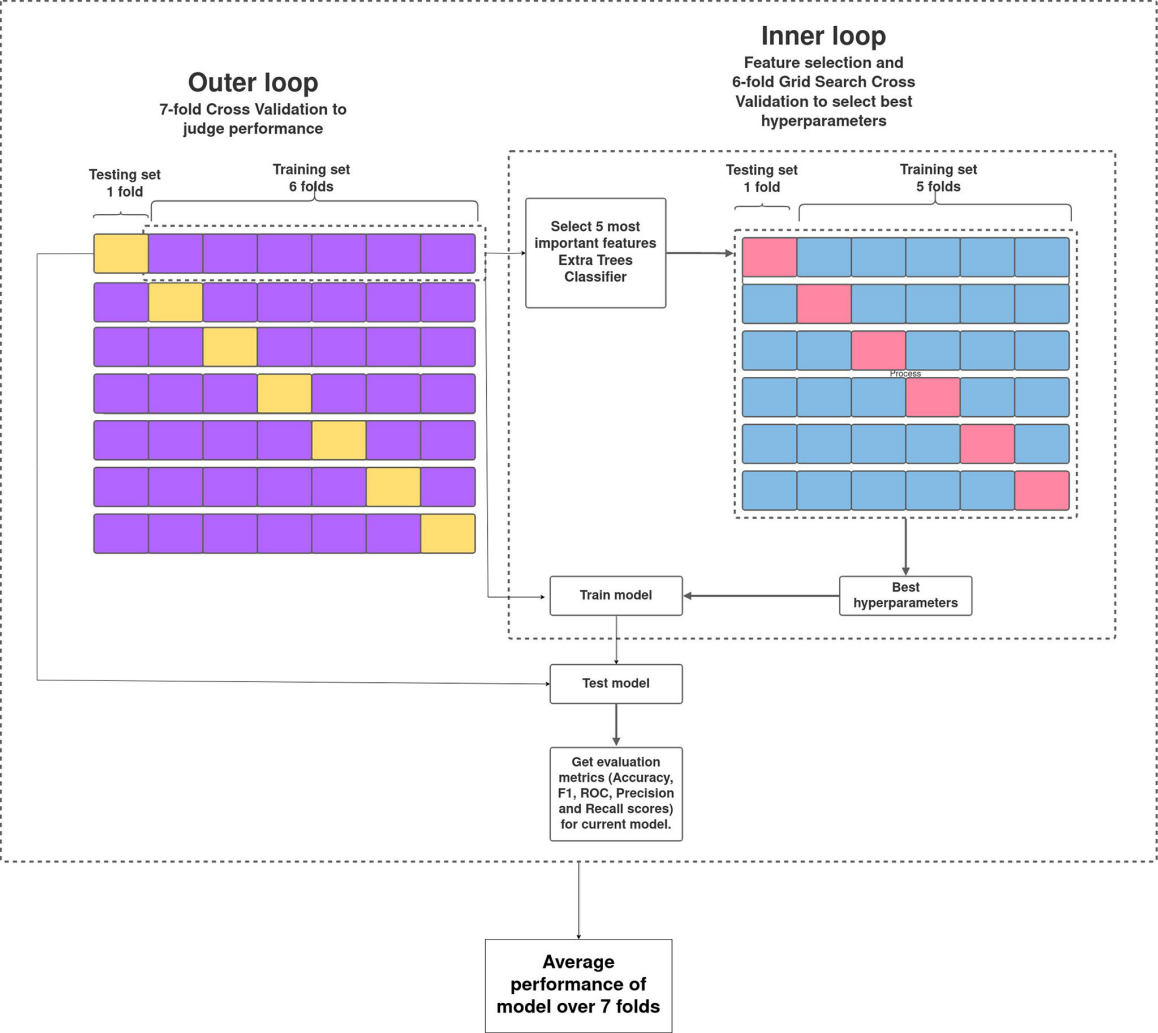
Overview of nested cross-validation. The outer loop is for feature selection and average performance of each model on the different training and testing folds, and the inner loop is for hyperparameter tuning using GridSearchCV.

The pipeline used to build the ML models in this study is described in **Figure 1**. Broadly, the curated dataset was divided into seven folds over seven iterations, six folds for training and one fold for testing in each iteration, thus covering the whole dataset. A value between 3 and 10 is generally selected for the number of folds, with a higher value leading to less bias. We chose to divide into seven folds because the dataset size is 175, 25 patients in each fold being ideal. This is the outer loop. In each iteration, all of the 31 different variables were passed into the function for building the ML models. Feature selection was done on the training folds using the Extra Trees Classifier, in which we selected the five most important features to train the model. For hyperparameter tuning, GridSearchCV, on the six training folds, was performed in the inner loop. ML algorithms of logistic regression, random forest, XGBoost, SVM, and multilayer perceptron were tested in this study. All five ML algorithms are trained on six folds and tested on the seventh, over seven iterations. After all seven iterations, the evaluation metrics were averaged out to report the final performance of the model. This method is known as nested cross-validation (CV). To avoid information leaking into the test set and overfitting of data, nested CV effectively uses a series of different train–test set splits in each iteration. Nested CV is the preferred way to evaluate and compare tuned ML models and has been used before in clinical settings (Gupta V. K. et al., 2021). **Figure 2** demonstrates the workflow of the nested CV algorithm. A detailed description of the steps used in building the models is given in the following sections.

### Data Normalization

Data normalization was performed using MinMaxScaler of scikit-learn library in Python, which performs a linear transformation on the original data. It was fitted on the training set and transformed on the training and test sets using the same fit.

### Feature Selection

Feature selection was done on the training folds using the ExtraTreesClassifier (Extremely Randomised Random Tree Classifier), which is present in the scikit-learn ensemble methods. It is a type of ensemble learning technique that accumulates multiple correlated trees in a forest and generates the classification result. Each feature was ordered in descending order according to its Gini importance, from which the five most important features were selected for the model.

### Hyperparameter Tuning

To perform hyperparameter tuning, grid search cross-validation over six folds of the current training set was performed. GridSearchCV is a package of scikit-learn that selects the best hyperparameters from a range of listed hyperparameters by trying all combinations of the values passed. They were scored based on the F1 scores (one of our major evaluation metrics).

### Evaluation Metrics

The following metrics were recorded to assess the predictive performance of the supervised models. The formulae for the calculation of all metrics are given below.

• **Accuracy score**: Accuracy is the fraction of predictions that the model got right.

$$\text{Accuracy}\;=\frac{\text{Number of correct predictions}}{\text{Total number of predictions}}$$

• **Precision**: Precision indicates what proportion of the positive predictions was correct. It is the number of correct positive results divided by the number of positive results predicted by the classifier.

$$\text{Precision}=\frac{\text{True positives}}{\text{True positives}+\text{False positives}}$$

• **Recall**: Recall denotes what proportion of the actual positives was identified correctly. It is the number of correct positive results divided by the number of all relevant samples.

$$\text{Recall}=\frac{\text{True positives}}{\text{True positives}+\text{False positives}}$$

• **F1 score**: The F1 score is the harmonic mean between precision and recall. The range for the F1 score is [0, 1]. It indicates how precise the classifier is (how many instances it classifies correctly) and how robust it is (it does not miss a significant number of instances).

$$\text{F}1\text{ Score}=2*\frac{\text{Precision}*\text{Recall}}{\text{Precision}+\text{Recall}}$$

• **ROC score**: The receiver operator characteristic (ROC) curve is a probability curve that plots the true-positive rate (what proportion of the positive class got identified correctly) against the false-positive rate (what proportion of the negative class got incorrectly classified) at various threshold values and essentially separates the “signal” from the “noise.” The area under the ROC curve (AUC) is a measure of the ability of a classifier to distinguish between classes and is used as a summary of the ROC curve.

## Results

### Patient Clinical and Demographic Characteristics

**Figure 3:**
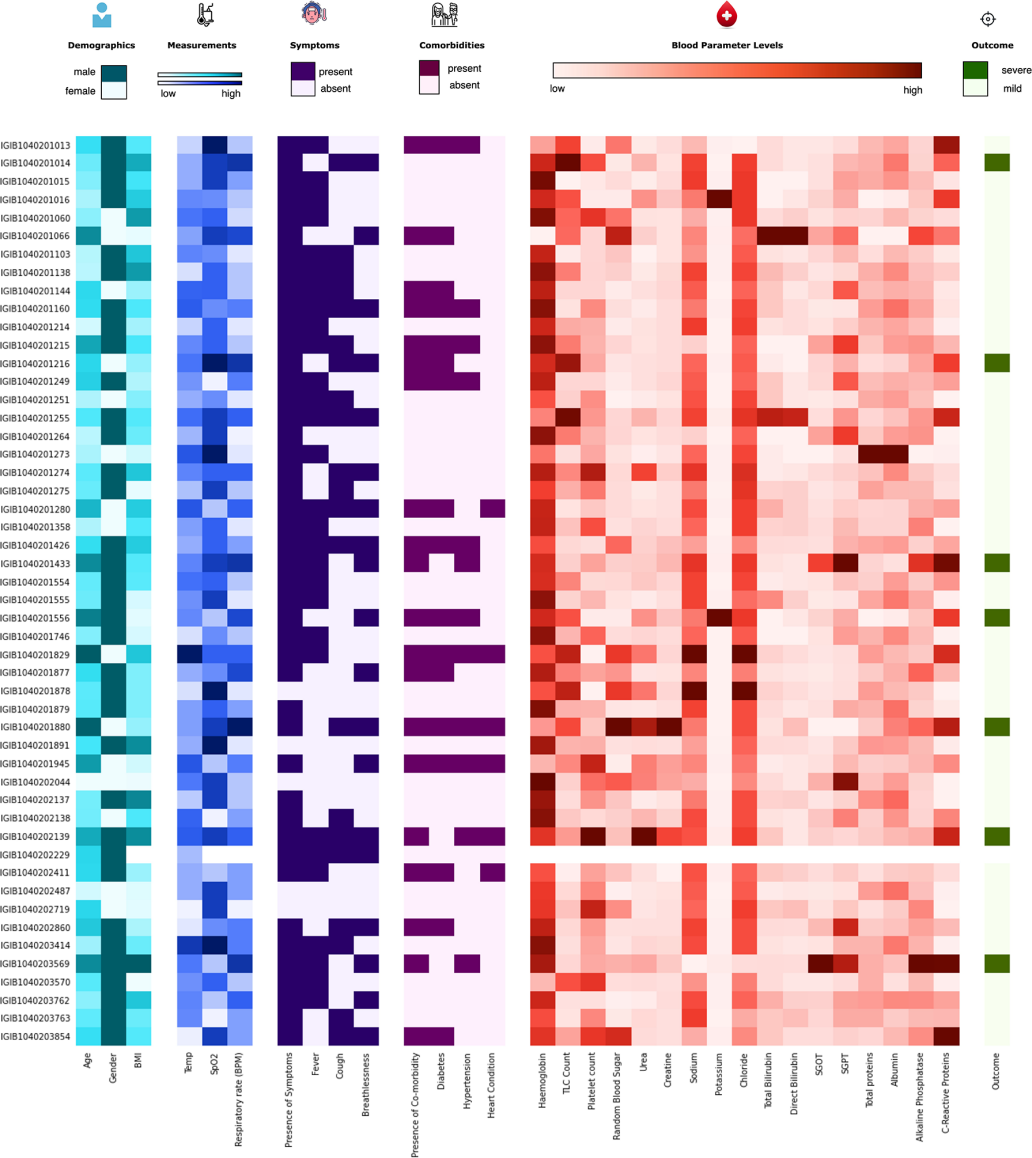
Distribution of the clinical parameters across severe acute respiratory syndrome coronavirus 2 (SARS-CoV-2) patients. The figure highlights the clinical heterogeneity across various clinical parameters in COVID-19 patients. These include demographic, clinical, and blood parameters and disease outcomes.

The initial dataset containing 257 patients was intensively curated to exclude patients with missing data on important parameters, resulting in a set comprising 175 patients with 31 different clinical parameters, which was used in the study. In this dataset, 24 patients were classified as severe and 151 as mild COVID-19 patients. The median age of the patients was 46 years, ranging from 5 to 92 years. Males comprise a higher percentage of the patients, constituting nearly 62%. The most common clinical features were fever (48.57%), cough (42.28%), and breathlessness (37.71%). The average respiratory rate was seen to be 14–34 breaths per minute (median = 20). The hematological parameters showed TLC counts from 2,500 to 25,200 cells/mm^3^, alkaline phosphatase median of 68, and CRP median of 12mg/L. A summary of the available parameters is presented in **Table 1**. Histograms of all continuous parameters are shown in **Supplementary Figures 1–3**. The diversity in the clinical presentations of the patients across various clinical parameters and the severity are illustrated in **Figure 3**.

**Table 1:** Clinical summary of patients positive for severe acute respiratory syndrome coronavirus 2 (SARS-CoV-2).

Patient features	Cohort (*n* = 175)	Mild (*n* = 151)	Severe (*n* = 24)	*p*-values
Age	46.00 (5–92)	43 (5–85)	70 (33–92)	**5.61E−06** ^a^
Gender				1.77E−01^b^
Female	68 (38.85%)	62 (35.42%)	6 (3.42%)	
Male	107 (61.14%)	89 (50.85%)	18 (10.28%)	
Temperature	98.10 (95.70–102.00)	98 (95.7–102.0)	98.6 (96.7–100.0)	1.86E−01^a^
BMI	22.70 (18.70–17.40)	22.8 (18.9–27.4)	22.4 (18.7–27.2)	3.32E−01^a^
SpO_2_	98.00 (94.00–100.00)	97.0 (94.0–100)	98.0 (94.0–100)	7.73E−01^a^
Respiratory rate (per minute)	20.00 (14.00–34.00)	18.0 (14.0–34.0)	28.0 (16.0–32.0)	**1.39E−17** ^a^
Presence of symptoms	144 (82.28%)	120 (68.57%)	24 (13.71%)	NA
Fever	85 (48.57%)	73 (41.71%)	12 (6.85%)	1.00E+00^b^
Cough	74 (42.28%)	56 (32%)	18 (10.28%)	**6.00E−04** ^b^
Breathlessness	66 (37.71%)	44 (25.14%)	12 (6.85%)	**5.29E−09** ^b^
Presence of comorbidities	84 (48.00%)	66 (37.71%)	18 (10.28%)	7.00E−03^b^
Hypertension	58 (33.14%)	45 (25.71%)	13 (7.42%)	3.30E−02^b^
Heart condition	21 (12.00%)	16 (9.14%)	5 (2.85%)	1.74E−01^b^
Diabetes	56 (32.00%)	45 (25.71%)	11 (6.28%)	1.57E−01^b^
Hemoglobin	13.70 (4.5–18.6)	13.60 (0–18.6)	13.95 (9.7–18.2)	6.84E−01^a^
TLC count	7,100.00 (2,500–25,200)	6,500.0 (2,500.0–24,700.0)	10,350.0 (5,200.0–25,200.0)	**4.21E−05** ^a^
Platelet count	214,000.00 (73,000.00–1,820,000)	214,000.0 (73,000.0–546,000.0)	208,000.0 (73,000.0–1,820,000.0)	5.20E−02^a^
Random blood sugar	114.00 (74.00–432.00)	111.0 (74.0–432.0)	125.0 (83.0–401.0)	6.59E−01^a^
Urea	26.70 (13.50–176.00)	25.9 (13.5–165.5)	48.0 (25.2–176.0)	**2.68E−05** ^a^
Creatine	1 (0.30–5800)	0.90 (0.3–5800.0)	1.05 (0.8–12.5)	6.99E−01^a^
Sodium	140 (124–159)	141.0 (124.0–159.0)	138.0 (127.0–146.0)	1.80E−02^a^
Potassium	4.30 (2.90–104.00)	4.30 (3.0–104.0)	4.45 (2.9–104.0)	1.18E−01^a^
Chloride	105.25 (1.10–125)	108.0 (1.1–125.0)	106.0 (88.0–111.0)	4.27E−01^a^
Total bilirubin	0.60 (0.30–4.20)	0.6 (0.3–4.2)	0.6 (0.3–2.4)	2.18E−01^a^
Direct bilirubin	0.20 (0.10–2.20)	0.2 (0.1–2.2)	0.2 (0.1–1.2)	6.21E−01^a^
SGOT	34.20 (0.20–474.20)	33.6 (0.4–285.0)	54.7 (0.2–474.2)	4.84E−03^a^
SGPT	31.20 (3.80–190)	31.0 (11.0–190.0)	40.7 (3.8–190.0)	1.86E−01^a^
Total proteins	6.40 (3.60–12.60)	6.5 (4.8–12.6)	6.0 (3.6–7.4)	8.00E−03^a^
Albumin	3.50 (2.60–6.60)	3.5 (2.6–6.5)	3.4 (2.7–6.6)	7.67E−01^a^
Alkaline phosphatase	68.00 (3.80–320.90)	67.8 (3.8–237.0)	75.1 (32.4–320.9)	1.28E−02^a^
C-reactive protein	12.00 (0.10–381)	5.67 (0.1–381.0)	126.25 (12.0–168.9)	**3.89E−11** ^a^

Outlier trimming was performed for SGOT, thus removing one outlier. Data are shown as median (IQR) or n (%). Significant parameters found after multiple correction testing are shown in bold.

TLC, total leukocyte count; SGOT, serum glutamic oxaloacetic transaminase; SGPT, serum glutamic pyruvic transaminase. The table highlights the spectrum of a multitude of clinical parameters across mild and severe patients and the p-values of every parameter in predicting disease severity.

a Point-biserial correlation.

b Fisher’s exact test.

### Symptoms Associated With Disease Severity

**Figure 4:**
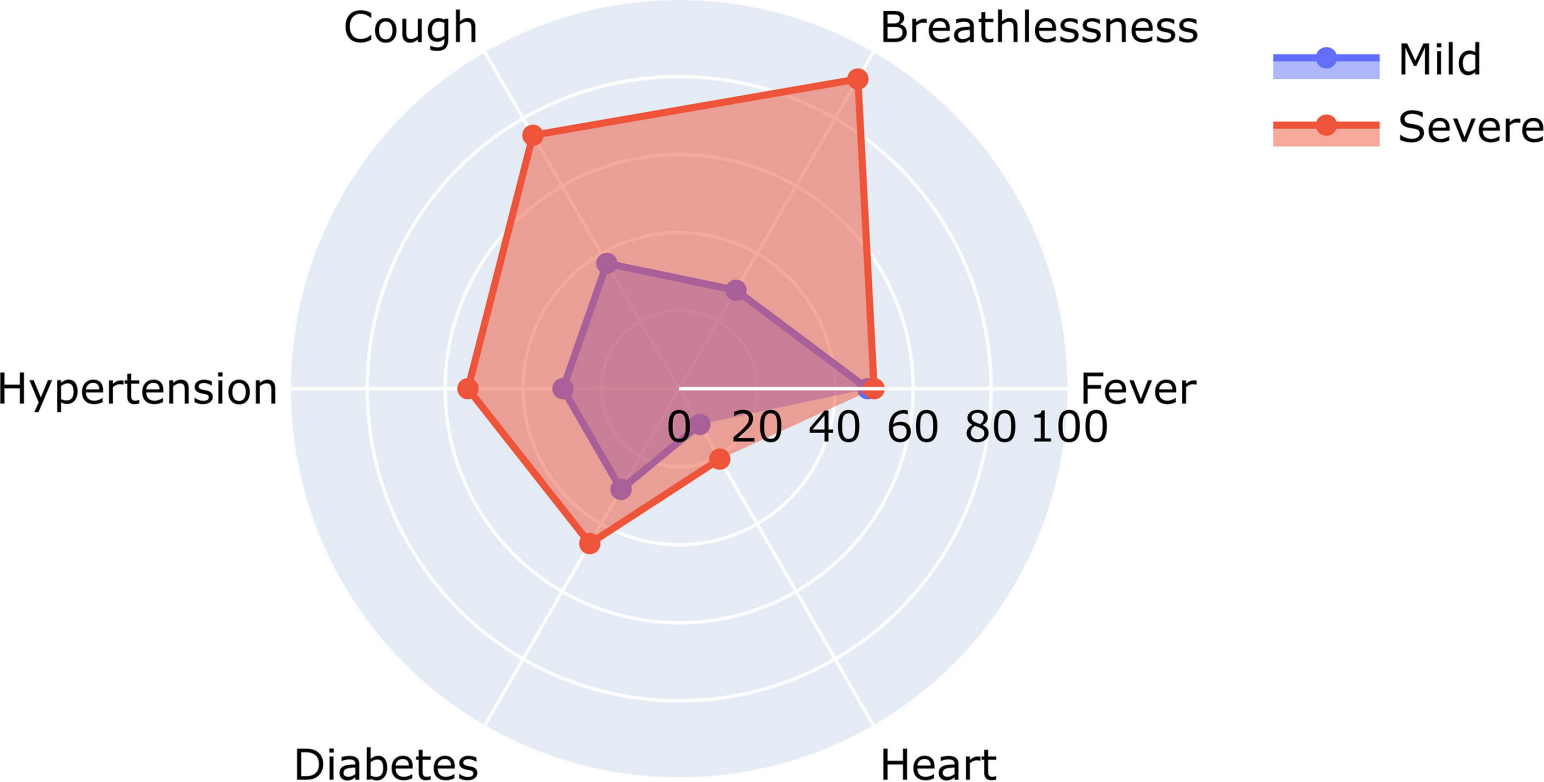
Relative abundance of symptoms in severe and mild coronavirus disease 2019 (COVID-19) patients.

To understand the significance of the clinical presentations of patients and the possibility of developing severe disease, we analyzed the statistical correlations of patients’ symptoms and comorbidities across mild and severe individuals. We observed symptoms such as breathlessness (*p* = 5.29E−09) and cough (*p* = 6.00E−04) to be significantly correlated with patients developing severe COVID-19. At the same time, comorbidities did not have a significant correlation with the disease severity. It may be mentioned that comorbidities are a diverse set of conditions that may have differential roles in modulating the disease. The differential abundance of these factors across mild and severe patients is highlighted in **Figure 4**.

**Figure 4** shows the differential number of patients included in our study with symptoms ranging from breathlessness, cough, fever, hypertension, diabetes, and heart disease.

A closer look at the clinical data also revealed that 22 out of 66 patients reporting breathlessness developed severe disease, while only 2 out of 109 patients not suffering from breathlessness developed severe disease. To further understand the significance of patients’ disease symptoms in delineating disease severity, we calculated the combined effect of symptoms on severity prediction. Unsurprisingly, we noted that the presence of all three symptoms—cough, fever, and breathlessness—increased the probability of contracting severe disease (**Table 2**).

**Table 2:** Correlation between severity and presence of multiple symptoms.

Fever	Cough	Breathlessness	No. of patients with these symptoms	Probability of severe outcome in our cohort
Absent	Absent	Absent	41	0
Absent	Absent	Present	13	0.23
Absent	Present	Absent	8	0
Absent	Present	Present	28	0.32
Present	Absent	Absent	37	0
Present	Absent	Present	10	0.3
Present	Present	Absent	23	0.09
Present	Present	Present	15	0.47

The table highlights the combinatorial effect of the most prominent disease symptoms and how it increases the probability of severe disease outcomes in the presence of multiple symptoms.

### Statistical Correlation Analysis of All Clinical Characteristics

**Figure 5:**
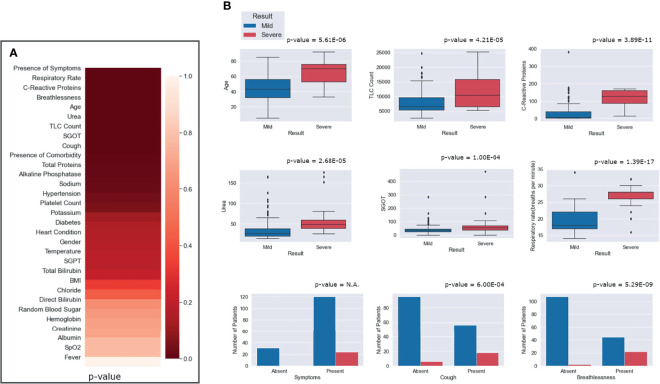
Statistical correlations of the clinical parameters and clinical severity. **(A)** Ranking of the parameters according to predictive significance. **(B)** Depiction of the differential abundance of clinical parameters across mild and severe patients. For the presence of any symptom, cough and breathlessness, the *p*-value is between mild and severe patients with respect to the presence and absence of symptoms.

To identify the clinical parameters associated with disease severity, statistical tests for correlation were used on the curated dataset of 31 parameters across 175 patients. We found that 13 parameters had a *p*-value <0.05, namely, age, alkaline phosphatase, breathlessness, CRP, SGOT, cough, hypertension, comorbidities, respiratory rate, sodium, TLC count, total proteins, and urea (**Table 1**). Upon further scrutiny of the significance threshold by performing multiple testing correlation, a *p*-value <0.0016 was considered significant, thus resulting in the identification of seven highly significant factors associated with severity. These factors are age (65.41 ± 16.75, *p* = 5.61E−06), breathlessness (*p* = 5.29E−09), CRP level (115.84 ± 45.81 mg/L, *p* = 3.89E−11), respiratory rate (26.5 ± 3.78 breaths/minute, *p* = 1.39E−17), TLC count (11,887.5 ± 6,378.108, *p* = 4.21E−05), coughing (*p* = 6.00E−04), and blood urea level (60.88 ± 42.21 mg/dl, *p* = 2.68E−05). We also noted that, in our dataset, there were no patients with severe clinical disease that were completely free of symptoms: cough, fever, and breathlessness. To understand the association of the clinical parameters and disease severity, bar graphs are plotted for all parameters in **Supplementary Figures 4–8**. The order of statistical significance of the 31 parameters are shown in **Figure 5A**, and significant correlations are shown in **Figure 5B**.

We observed that patients above the age of 60 years were more prone toward having severe COVID-19. It is important to note that only 8 out of 130 people below 60 years had severe disease, while 16 out of 45 people above 60 years had severe disease outcomes. **Figure 5B** highlights that, for patients with a respiratory rate above 25 breaths/minute, the outcome was most likely to be severe. Similarly, it was observed that a CRP level above 100 mg/L was correlated with severity, albeit with a few outliers.

### Machine Learning Model Development

Five different ML algorithms were used to develop viable predictive models for disease severity. All the models were tested using nested CV and their average performance metrics reported. For the final ROC curve, the ROC curve for each iteration of nested CV was plotted and averaged to obtain the final curves (**Supplementary Figure 9**).

**Figure 6:**
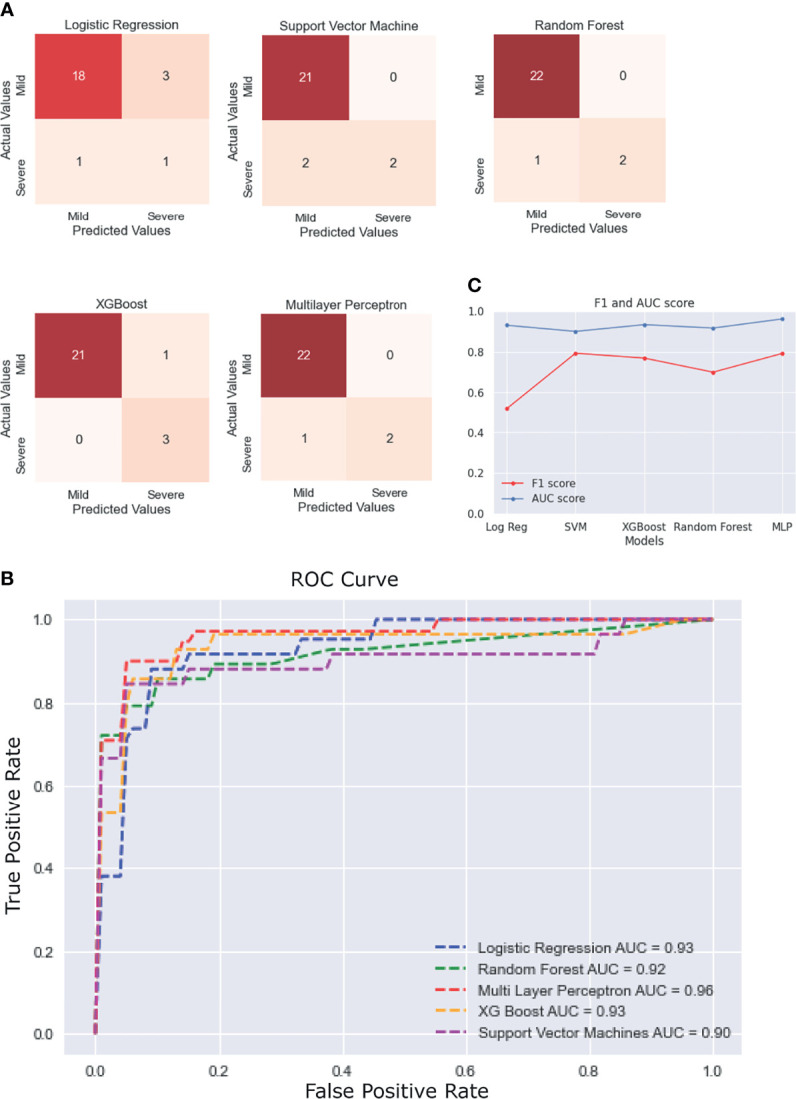
Performance of the different machine learning (ML) models. **(A)** Confusion matrices of the last fold in the nested cross-validation (CV) for each model. **(B)** Plot of the average receiver operating characteristic (ROC) curve of each model. **(C)** Line plot for the F1 and area under the curve (AUC) scores for each model.

The statistical results previously obtained tallied with the features that were selected for most models using ExtraTrees Classifier. Every model showed more than 90% accuracy, but their performance was judged by comparing the ROC AUC scores and F1 scores, as these measures are better suited for an imbalanced dataset. It was observed that most models performed similarly to each other with respect to the ROC-AUC scores (in the range of 0.90–0.93), but multilayer perceptron stood out with an ROC score of 0.96. In terms of the F1 scores, SVM performed the best (F1 score = 0.793), followed closely by multilayer perceptron (F1 score = 0.791), with a difference of just 0.002. It must be noted that the results would vary slightly with each test run, so it can be said that, for our test run, multilayer perceptron performed the best overall with the second best F1 score of 0.791 (very close to that of the first being 0.793) and the best ROC score of 0.96. The scores of each model across various performance metrics are shown in **Supplementary Table 1**. A detailed comparative report of the model performances is shown in **Figure 6**, with the confusion matrices, ROC curve, and F1 scores of each model.

### SARS-CoV-2 Phylogenetic Analysis

**Figure 7:**
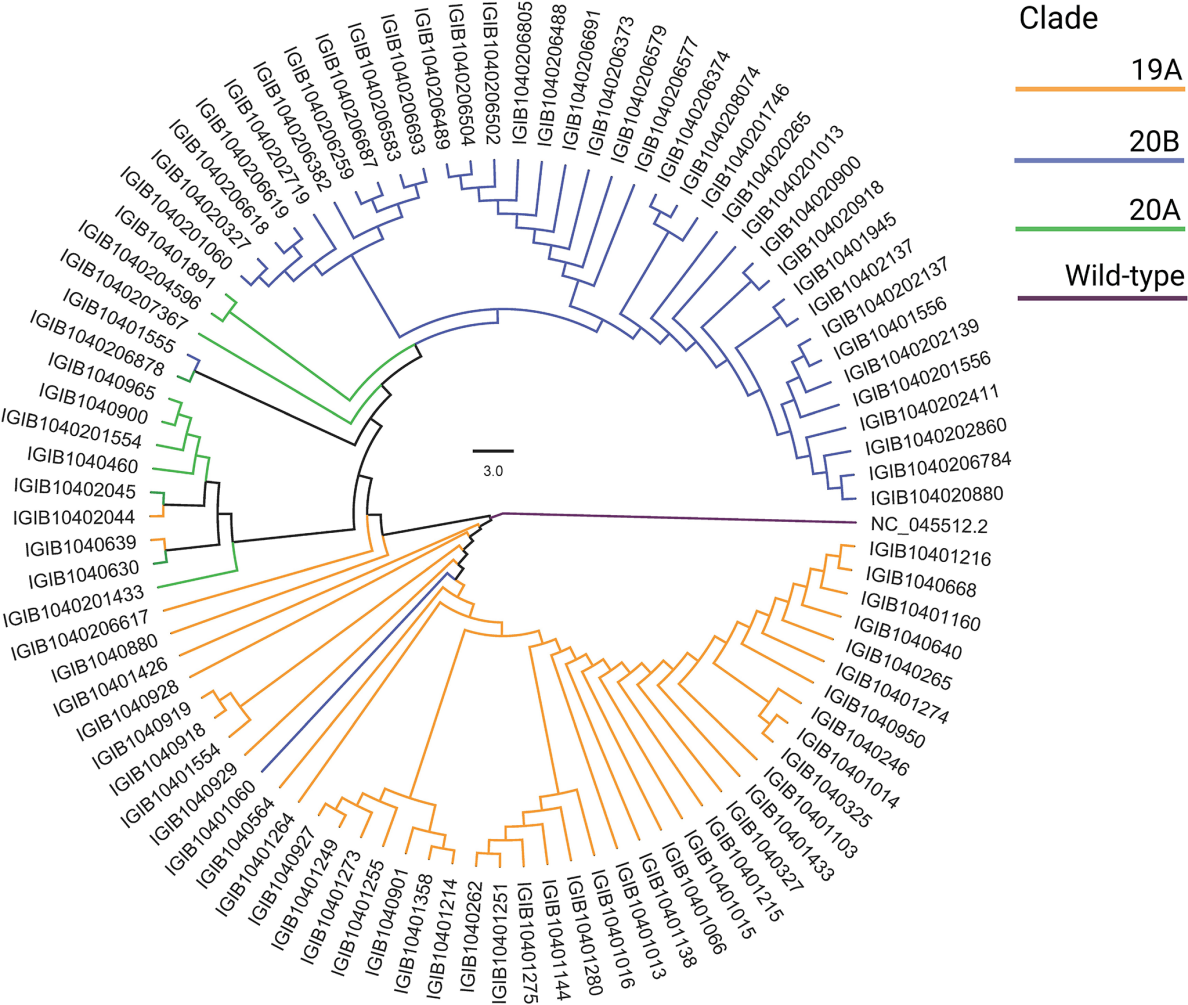
Phylogenetic analysis of the severe acute respiratory syndrome coronavirus 2 (SARS-CoV-2) genomes. The distribution of SARS-CoV-2 clades among 92 coronavirus disease 2019 (COVID-19) patients compared with the wild-type strain is shown.

SARS-CoV-2-positive nasopharyngeal RNA samples from 92 patients out of 257 in the patient cohort with sufficient quantity and quality of RNA were available for viral genome sequencing. To understand the SARS-CoV-2 genomic diversity in our patient cohort, genome sequencing and analysis were performed on these RNA samples. Phylogenetic analysis showed that the majority of the samples belonged to clades 19A (47.8%), 20A (11.9%), and 20B (40.2%) (**Figure 7**). This is consistent with the SARS-CoV-2 genomic surveillance observations in India during a similar period of the pandemic (Banu et al., 2020; Kumar et al., 2020; Joshi et al., 2021). The clades and genome coverage of all sequenced patient samples are listed in **Supplementary Table 2**.

### SARS-CoV-2 Mutation Analysis

**Figure 8:**
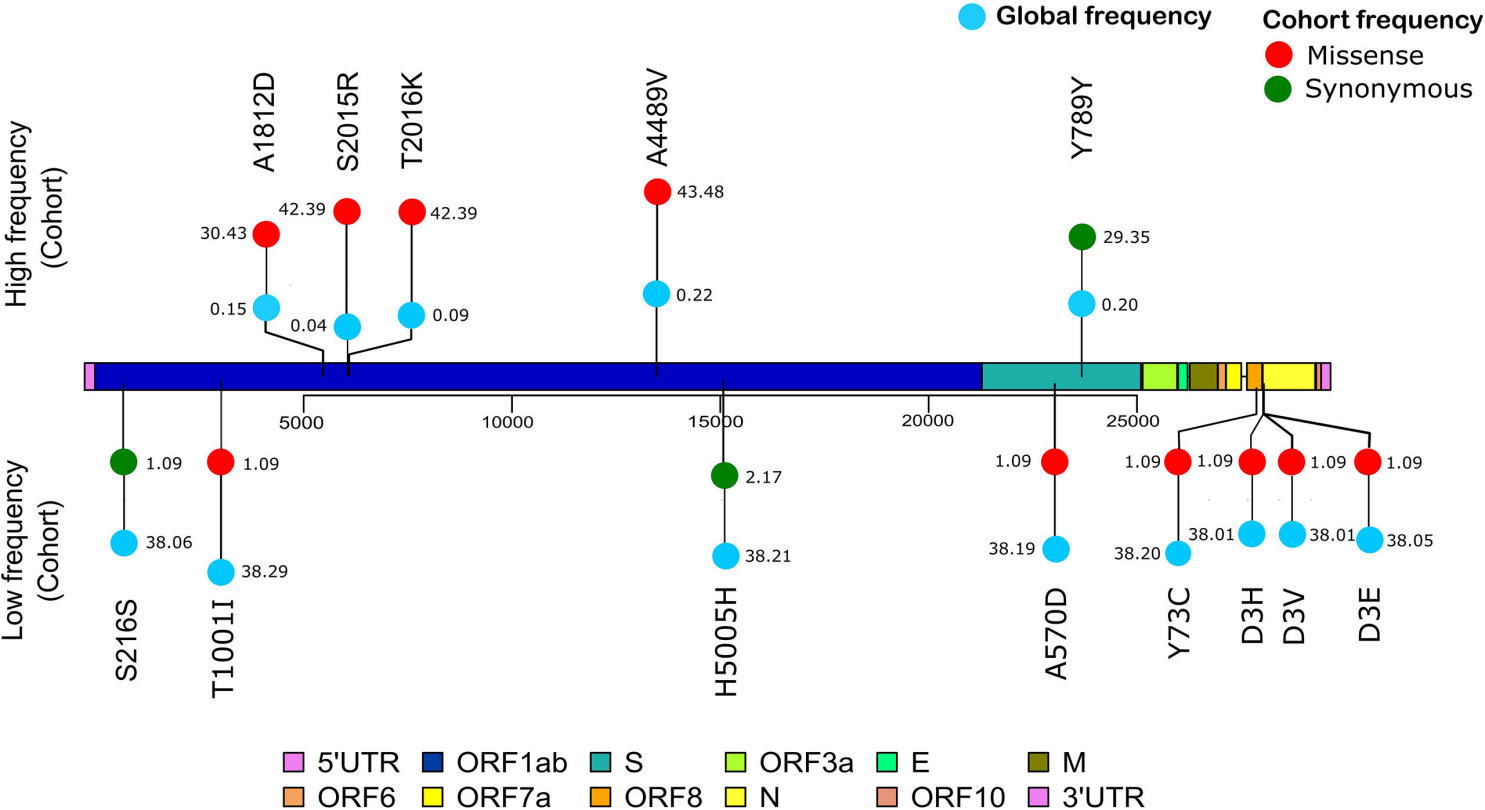
Mutation frequency analysis with frequency flip. The frequency of mutations with significant frequency differences between the global and our study cohort are shown. Global frequency is represented as *blue circles* and the missense mutation frequency in our cohort is shown as *red circles*. Synonymous mutation frequencies in our cohort, S216S (C913T), H5005H (C15279T), and Y789Y (C23929T), are shown as *green circles*.

To further observe the diversity of mutations captured in our samples, we performed mutational analysis vis-a-vis comparison with the global frequency and genomic distribution of the observed mutations. Individual mutation level analysis revealed 422 unique mutations in our sample set. Further analysis toward the global frequency of all these mutations during SARS-CoV-2 surveillance with 21,51,254 globally shared sequence data revealed that eight mutations present in low frequencies (lower quartile) in our dataset are now present in higher frequencies (>35%) when compared with the global mutational frequency data. The evolutionary selection of these mutations over the period of the pandemic (from May 2020 to Sept 2021) and their presence in the VOCs and VOIs indicate their potential functional significance. Orthogonally, we noticed five highly frequent mutations in our dataset (upper quartile) that are now present in very low frequency (<1%) in current global data (**Figure 8**). These mutations can possibly have a detrimental effect on the improved transmission characteristics observed in the latest variants spreading across the globe. We also noticed six mutations in our dataset (D614G, P4715L, R203R, R203K, C15279T, and Q57H) that have currently been designated as clade-defining mutations of the VOCs and VOIs of SARS-CoV-2, namely, 20I (Alpha), 21A (Delta), 21B (Kappa), and 20H (Beta). All mutations were then annotated on the SARS-CoV-2 reference genome to identify the presence of the mutations in different regions of the SARS-CoV-2 genome. We observed 67% of the mutations to be present in the ORF1ab region and 11.76% of the mutations to be present in the spike region of the SARS-CoV-2 genome. It is interesting to note that, due to the size of the SARS-CoV-2 spike region being around one-fifth of the ORF1ab region, we saw equal mutation rates in the spike and ORF1ab regions, which were around 1.31 and 1.34, respectively. We also observed a significant number of mutations present in other regions of the genome (**Supplementary Table 3**).

## Discussion

COVID-19 has spread around the globe with rapidly emerging VOCs and VOIs reducing the efficacy of global vaccination efforts, which has transformed the pandemic to an ever-looming threat to global healthcare. Therefore, meaningful surveillance efforts and an efficient, applicable COVID-19 risk prediction model based on the Indian cohort are warranted. In this study, we analyzed 175 patient clinical data with 31 different parameters, which included vital signs and patient demographic data on oxygen saturation, respiratory rate, BMI, age, gender, comorbidities, and respiratory support levels. Detailed blood test reports pertaining to the levels of CRP, IL-6, TLC, D-dimer, and lactate dehydrogenase were also included in the study. We observed that symptoms such as breathlessness and cough segregated toward severe outcomes in due course of disease progression. The significance of these symptoms has been previously reported by different studies (Burke et al., 2020; Ioannou et al., 2020), whereas the presence of symptoms such as cough, fever, and breathlessness together significantly increased the probability of the patient developing severe COVID-19. This has been shown in other cohorts, but not in India (Ioannou et al., 2020). To further improve the identification of risk factors associated with COVID-19, we analyzed every biochemical test parameter for its severity predictive significance. We observed that seven parameters—age above 60 years, breathlessness, CRP level above 100 mg/L, cough, respiratory rate above 25 breaths/minute, TLC count above 10,000, and blood urea level above 40 mg/dl—showed a statistically significant (*p* < 0.0016) correlation with disease severity. Some of these parameters have been individually reported to be of predictive significance in COVID-19: CRP levels (Ali, 2020), blood urea levels signifying kidney involvement (Cheng et al., 2020b), TLC (Zhao et al., 2020), and old age (Mueller et al., 2020). Using these factors in a combined manner can help to assess the risk of patients developing severe disease. Although due to the relatively small patient cohort in the study, albeit detailed clinical parameters, it would be important to validate the findings in a larger Indian dataset to further iterate the findings and highlight the significance of these clinical parameters in predicting COVID-19 severity and clinical outcomes.

To enable a robust risk stratification procedure to be implemented in clinical settings, five ML models for risk stratification were developed. Herein, we observed that multilayer perceptron outperformed all other models, with an ROC score 0.96 and an F1 score 0.791. Upon close observation of the characteristics of the other models, it became clear that a simpler model such as logistic regression (F1 score = 0.51) cannot capture the complexity of the dataset, even after selecting the best features and tuning the hyperparameters. In various other studies with similar models (Prakash, 2020; Gupta V. K. et al., 2021), it was observed that logistic regression underperformed. In our study, random forest, multilayer perceptron, XGBoost, and SVM performed much better and had similar scores, so we can use the results of the four combined to arrive at a prediction. The only plausible limitation of the approach is the relatively small sample size, which may limit how well the models built here generalize and the lack of repeatability of the ML-generated results. However, it is important to mention that the ML pipeline described adapts to and can be applied as a method for larger datasets, when available, making it a novel approach to the problem.

In our genomic analysis of 92 patients, we identified clades 19A, 20A, and 20B. To help stratify the SARS-CoV-2 mutations with respect to potential functional significance, we accessed the current global frequency of all 422 mutations identified in our dataset. Herein, we observed a few mutations that have a significant frequency flip for their occurrence. We noticed eight mutations present in extremely low frequencies in our dataset (*n* ≤ 2 patients), but were highly abundant in the global dataset (>35%). All of these mutations are now seen predominantly in VOC 20I Alpha. The mutations C3267T (T1001I) (Public Health England, 2021; Castonguay et al., 2021; Srivastava et al., 2021), A28111G (Y73C) (Public Health England, 2021, C23271A (A570D) (Public Health England, 2021), C913T (S216S) (Castonguay et al., 2021), T28282A (D3E) (He et al., 2021), G28280C (D3H) (Castonguay et al., 2021; He et al., 2021), and A28281T (D3V) (Castonguay et al., 2021) are reported in 20I Alpha clade. Of these, C15279T (H5005H) is a clade-defining mutation for 20I (Hadfield et al., 2018). The identification of these mutations associated with 20I in the Indian cohorts around the period from July to September 2020 is an interesting finding for further evolutionary analysis of clade 20I, as it is believed to have originated from the UK where it was first discovered on September 20, 2020 (Public Health England, 2021). Orthogonally, five of the mutations highly abundant in our dataset were seen to be sparsely present in the global data (<1% frequency). The literature reviews of these mutations—C13730T (A4489V) (Banu et al., 2020; Alai et al., 2021), (C6312A) T2016K (Banu et al., 2020; Sarkar et al., 2021), C6310A (S2015R) (Kumar et al., 2020), C5700A (A1812D) (Joshi et al., 2021; Srivastava et al., 2021), and C23929T (Y789Y) (Banu et al., 2020; Joshi et al., 2021; Sarkar et al., 2021)—reiterate this finding as they showed that all of these mutations were highly prevalent during the initial phase of the pandemic in India as a part of clades 19A, 20A, and 20B, whereas these mutations are not a part of the currently circulating clades, even in India, such as 21A (Delta) (Dhar et al., 2021; Shastri et al., 2021). This possibly indicates the detrimental effect of these mutations in viral transmission characteristics. Our effort toward mutational analysis beyond strain identification of the SARS-CoV-2 variants can provide epidemiological context to help prioritize SARS-CoV-2 mutations for functional analysis. However, cohort-specific genomic analysis provides valuable insights into the evolving genomic characteristics of the virus. Further validation toward the prevalence of the identified mutations can be done by continuous monitoring of the regional genomic trends of COVID-19 patients at the same time point and beyond.

**Figure 9:**
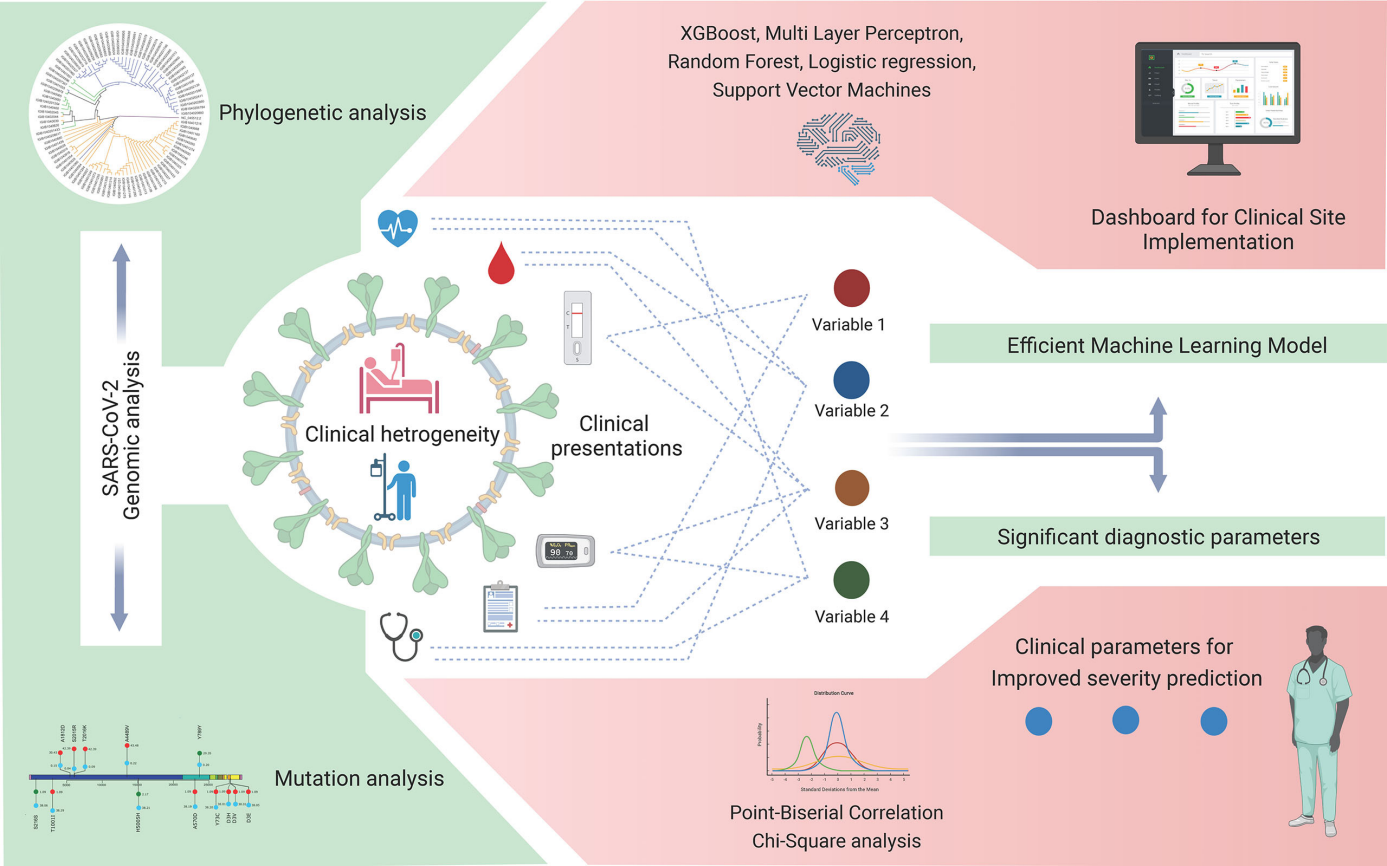
Severe acute respiratory syndrome coronavirus 2 (SARS-CoV-2) genomic and coronavirus disease 2019 (COVID-19) clinical data analyses for variant detection and patient severity classification. Clinical data analysis using a machine learning model for the identification of crucial clinical variables for risk stratification and potential development of a stand-alone dashboard for healthcare implementation.

An accurate risk stratification model for segregating disease-specific patient populations based on detailed clinical parameters can help in the rapid screening and resource allocation in healthcare facilities. Cohort-specific changes can be present between patients of different ethnicities (Ali, 2020), therefore necessitating the development of cohort-specific models for country-specific healthcare settings. Our study hereby provides an approach and method to converge the diversity of the clinical variables observed in the early phases of COVID-19 into a few consequential diagnostic variables for severity prediction. This ML model, upon further validation in larger patient cohorts across India, can be implemented by a clinician using an interactive dashboard at a healthcare facility in the future. A brief outline of the approach and future perspectives of this study are summarized graphically in **Figure 9**.

## Conclusion

The findings of this study highlight the integrative analysis of the diverse clinical data, SARS-CoV-2 genomic mutations, its future relevance when compared with the global frequency of the mutations, and the use of ML to reduce the dimensionality of the data in order to identify key features associated with disease severity. The findings regarding the low-frequency mutations being part of future VOCs and VOIs provide a framework for closely monitoring the low-frequency mutations for their future functional importance in transmission and immune escape.

## Data Availability Statement

The clinical dataset collected and analyzed as a part of this study is attached as **Supplementary Document 1**. The SARS-CoV-2 Genomic data used in the study has been uploaded to GISAID with accession numbers EPI_ISL_4503527 to EPI_ISL_450361, and EPI_ISL_4518814. The code for the NestedCV method used in the study for building and analysing various risk prediction models is available at https://github.com/INGEN-HOPE/NestedCV.

## Ethics Statement

The studies involving human participants were reviewed and approved by the CSIR-Institute of Genomics and Integrative Biology (CSIR-IGIB). Written informed consent to participate in this study was provided by the participants’ legal guardian/next of kin.

## Author Contributions

SB, AP, AK, RM, JV, PD and PC performed the analysis. SB, AP, AK, and RP wrote the manuscript. AK and RP designed, conceptualized, implemented, and coordinated the study, along with inferences of the results, and wrote the manuscript. SS, RK, and MJ shared the clinical samples and clinical data. All authors contributed to the article and approved the final version.

## Funding

This research was funded by the Fondation Botnar (project code: CLP-0031), Indo-US Science and Technology Forum (IUSSTF) (project code: CLP-0033), Intel (project code: CLP-0034), and Bill and Melinda Gates Foundation (BMGF) (project code: CLP-0036). This study received funding from Intel in addition to other funding support. The funders, inclusive of Intel, were not involved in the study design, collection, analysis, interpretation of data, the writing of this article, or the decision to submit it for publication.

## Conflict of Interest

The authors declare that the research was conducted in the absence of any commercial or financial relationships that could be construed as a potential conflict of interest.

## Publisher’s Note

All claims expressed in this article are solely those of the authors and do not necessarily represent those of their affiliated organizations, or those of the publisher, the editors and the reviewers. Any product that may be evaluated in this article, or claim that may be made by its manufacturer, is not guaranteed or endorsed by the publisher.
