# Supplementary material for: Learning From Biological and Computational Machines: Importance of SARS-CoV-2 Genomic Surveillance, Mutations and Risk Stratification
Source: Front Cell Infect Microbiol. 2021 Dec 24;11:783961. doi: 10.3389/fcimb.2021.783961 (PMC8762993; doi:10.3389/fcimb.2021.783961)
Supplement: Supplementary file 1 [file DataSheet_1.pdf]

Learning from Biological and Computational machines: importance of SARS-CoV-2 genomic surveillance, mutations and risk stratification

Shikha Bhat[1,#], Anuradha Pandey[1,#], Akshay Kanakan[1,#,*], Ranjeet Maurya[1,2], Janani Srinivasa Vasudevan[1], Priti Devi[1,2], Partha Chattopadhyay[1,2], Shimpa Sharma[3], Rajesh J. Khyalappa[3], Meghnad G. Joshi[3], Rajesh Pandey[1,2,*]

[1]INtegrative GENomics of HOst-PathogEn (INGEN-HOPE) laboratory, CSIR-Institute of Genomics and Integrative Biology (CSIR-IGIB), Mall Road, Delhi-110007, India.
[2]Academy of Scientific and Innovative Research (AcSIR), Ghaziabad-201002, India.
[3]Dr. D. Y. Patil Medical College, Kolhapur, Maharashtra-416006.

[#]Joint First Authors

[*]Co-corresponding authors

Contact Details:
Rajesh Pandey, PhD
Principal Scientist,
INtegrative GENomics of HOst-PathogEn (INGEN-HOPE) laboratory,
CSIR-Institute of Genomics and Integrative Biology (CSIR-IGIB),
North Campus, Near Jubilee Hall, Mall Road, Delhi-110007, India.
www.igib.res.in
Contact: rajeshp@igib.in; Tel.: +91 9811029551

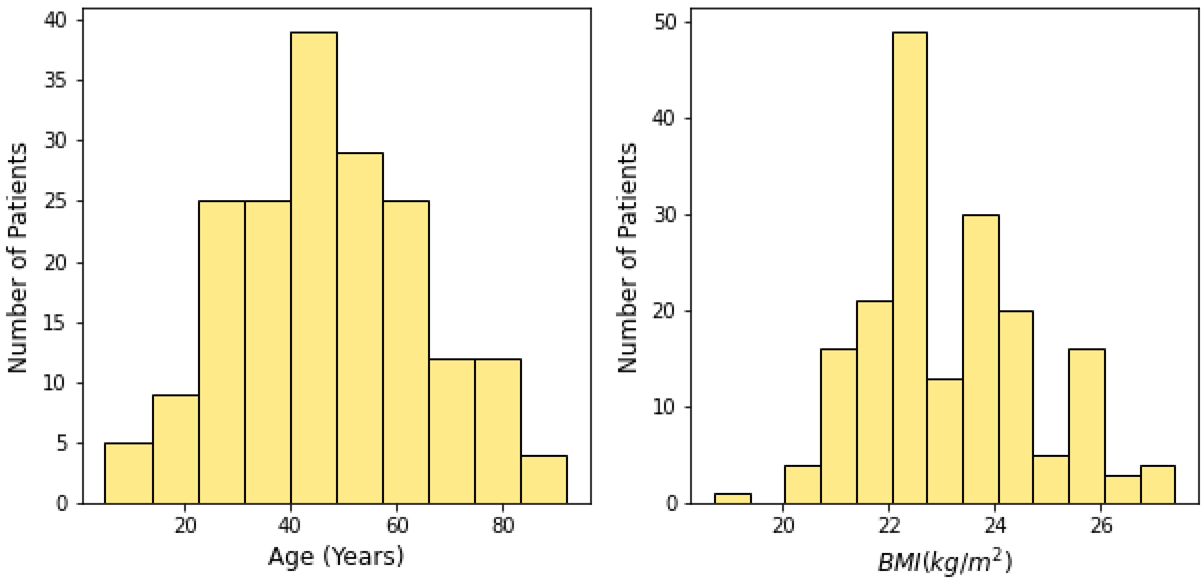

Supplementary Figure 1: Depicting the distribution of patients across continuous demographic parameters in COVID-19.

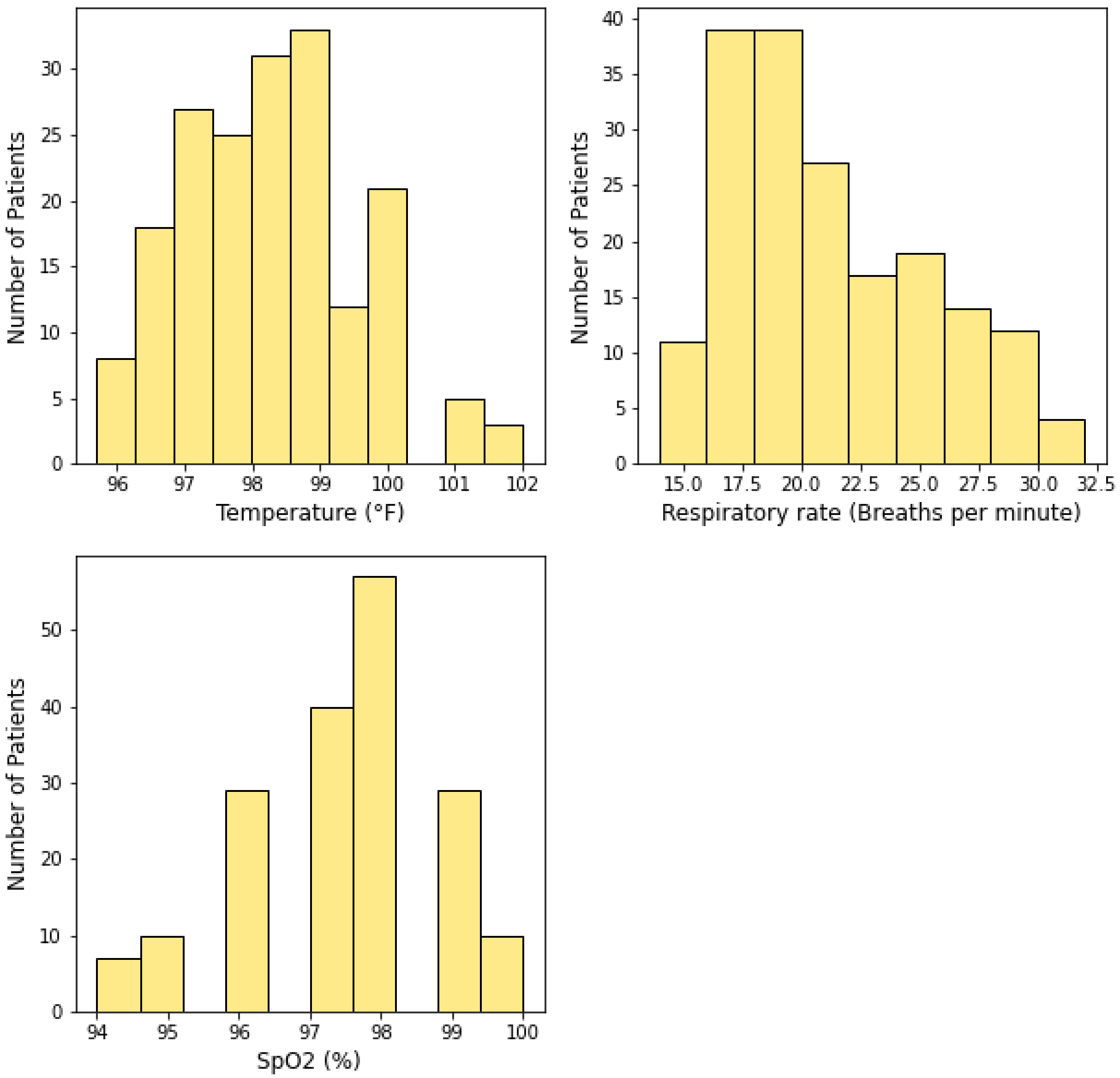

Supplementary Figure 2: Depicting the distribution of patients across continuous physiological measurements in COVID-19

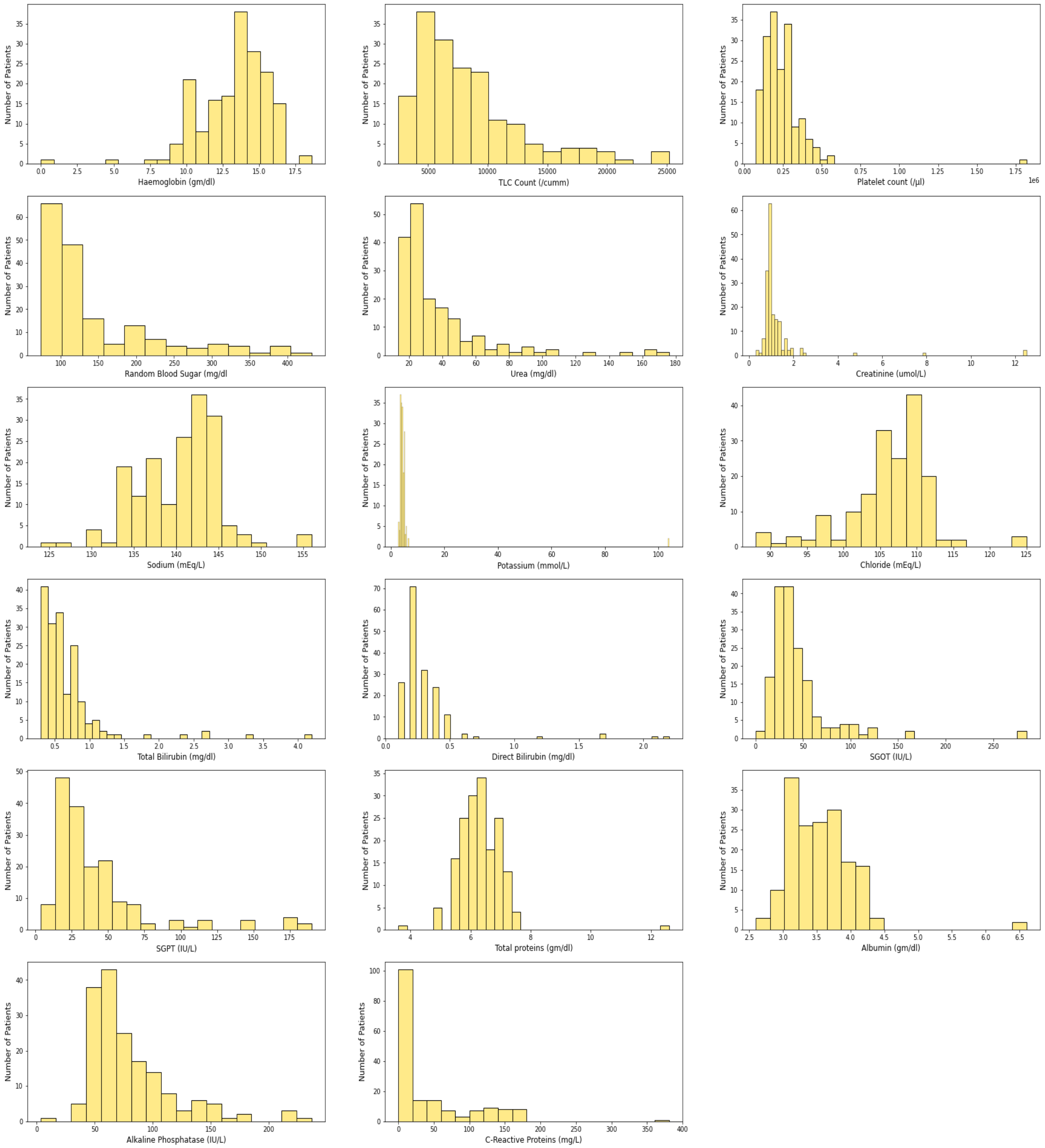

Supplementary Figure 3: Depicting the distribution of patients across continuous blood parameters in COVID-19.

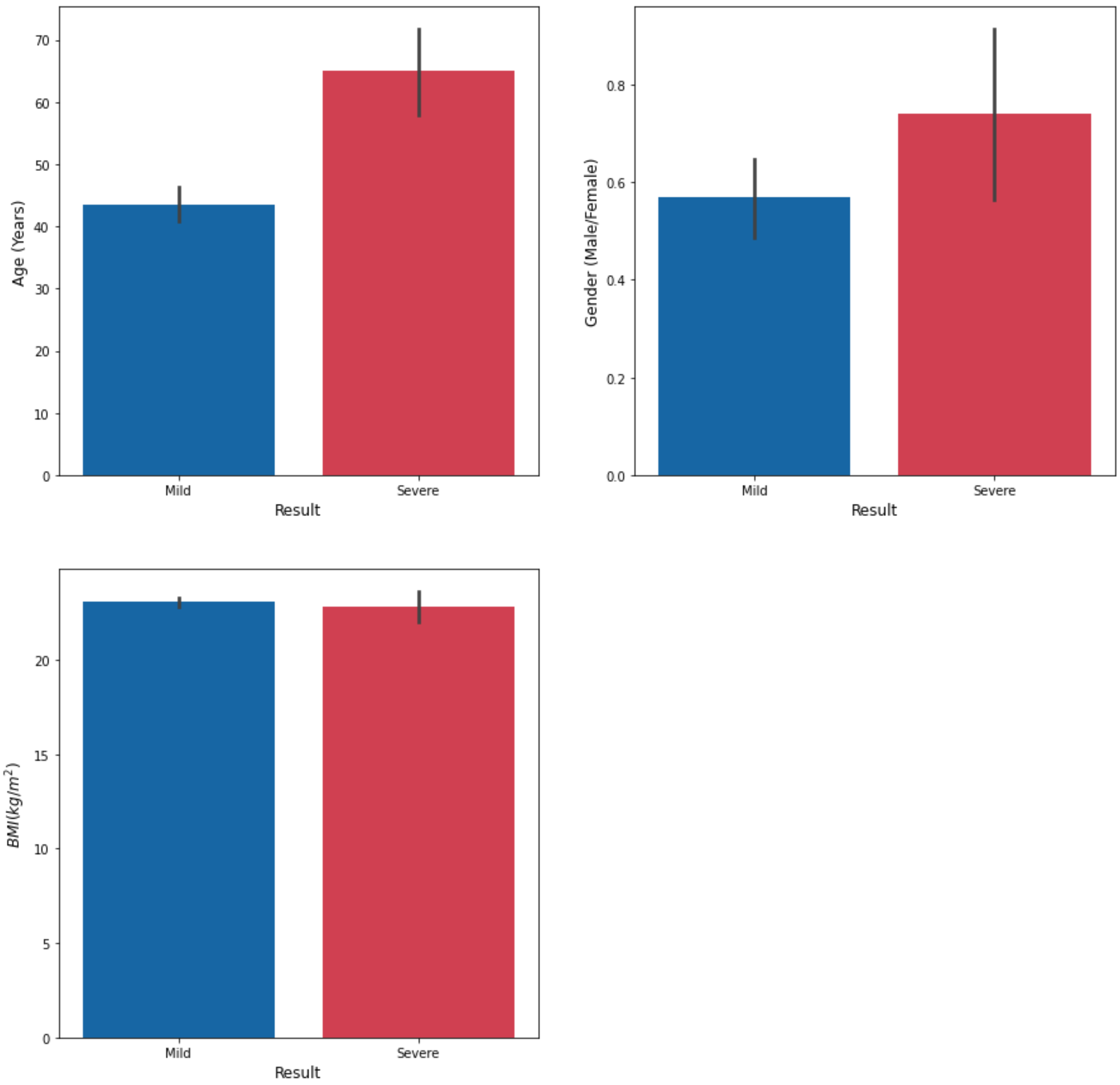

Supplementary Figure 4: Depicting the difference in demographics parameters across mild and severe COVID-19 patients. Black line showing standard deviation of bar plot.

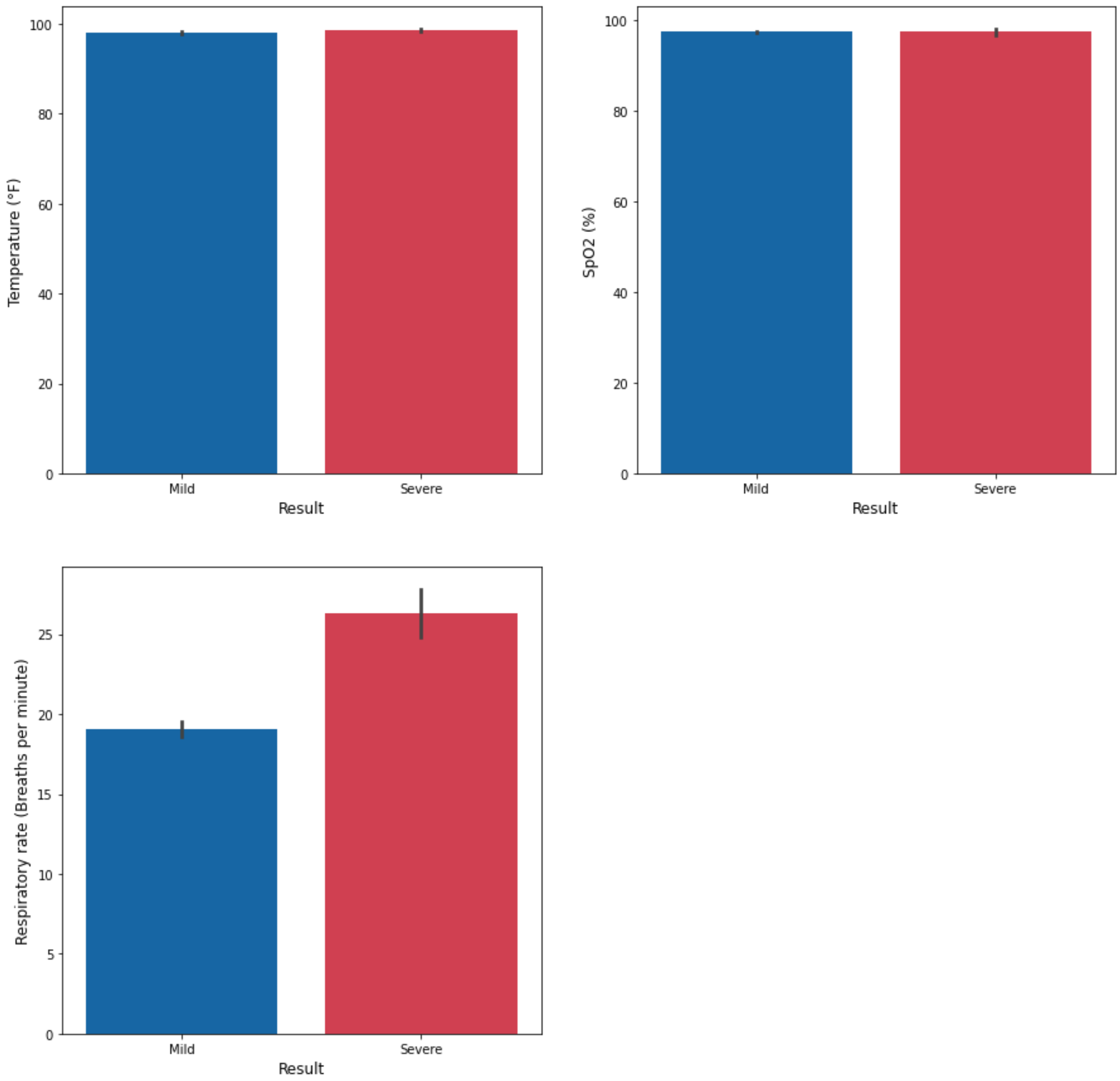

Supplementary Figure 5: Depicting the difference in physiological clinical measurements between mild and severe COVID-19 patients. Black line showing standard deviation of bar plot.

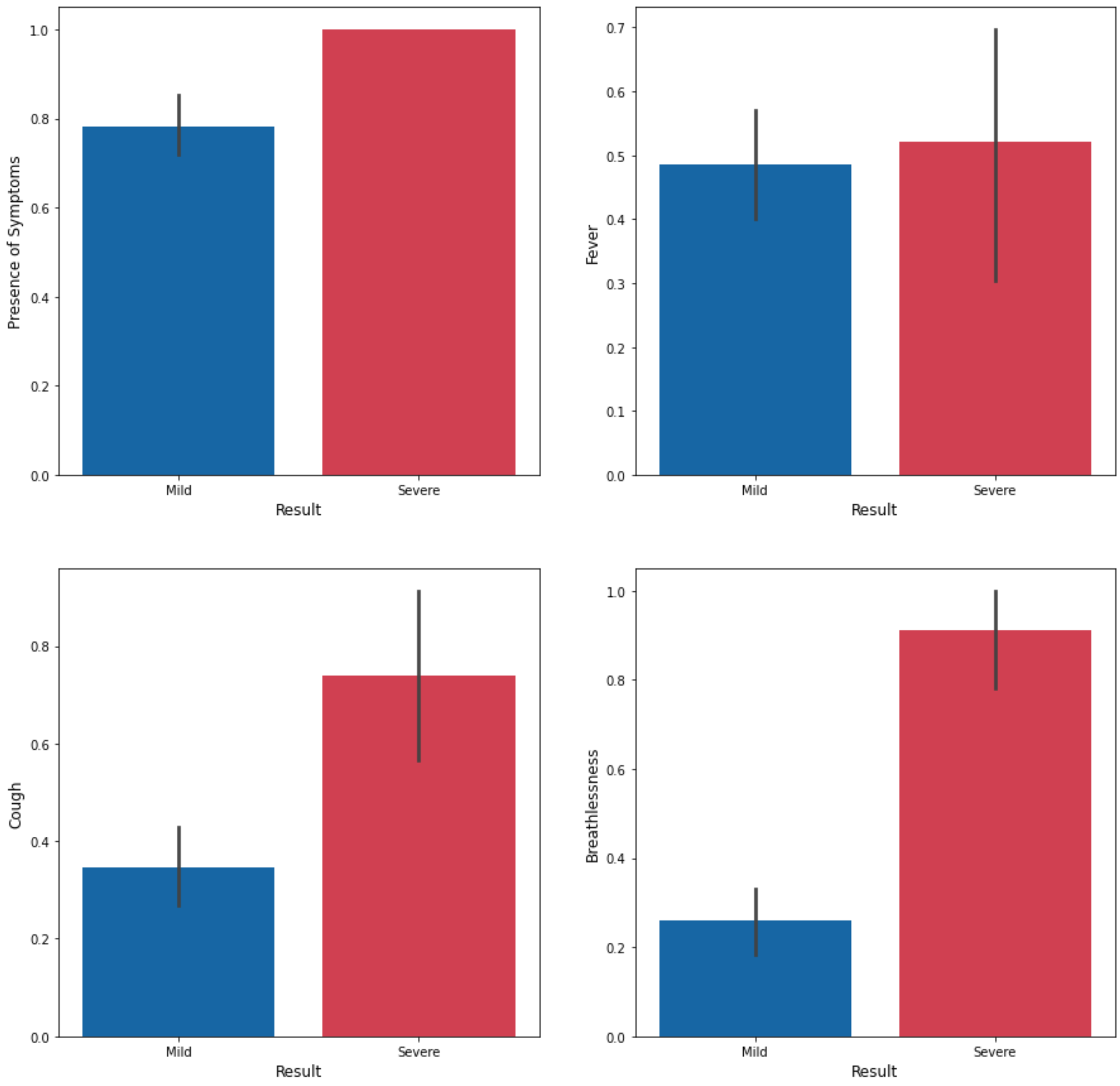

Supplementary Figure 6: Depicting the difference in percentages of mild and severe COVID-19 patients reporting various symptoms. Black line showing standard deviation of bar plot.

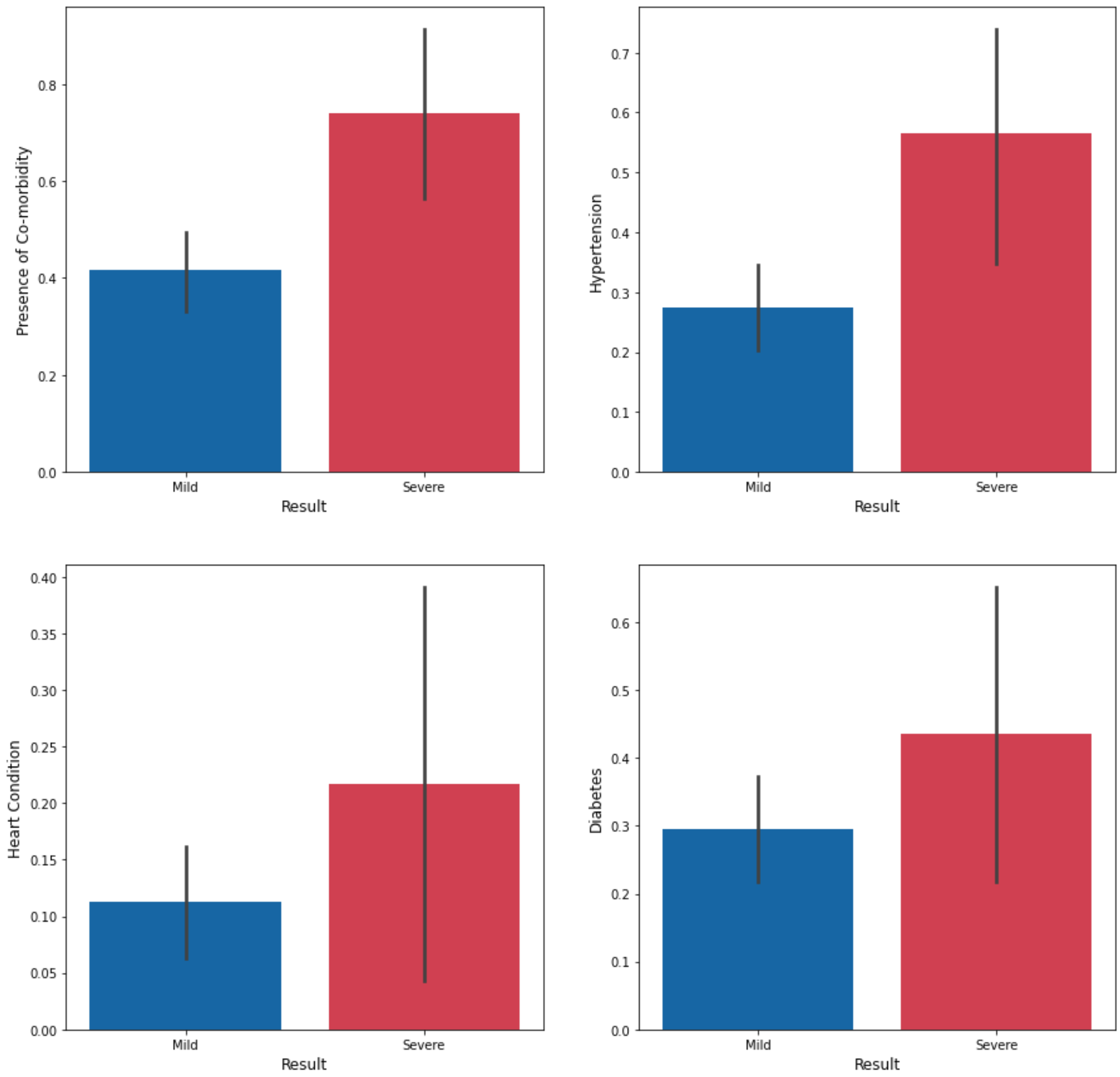

Supplementary Figure 7: Depicting the difference in percentages of mild and severe COVID-19 patients with pre-existing comorbidities. Black line showing standard deviation of bar plot.

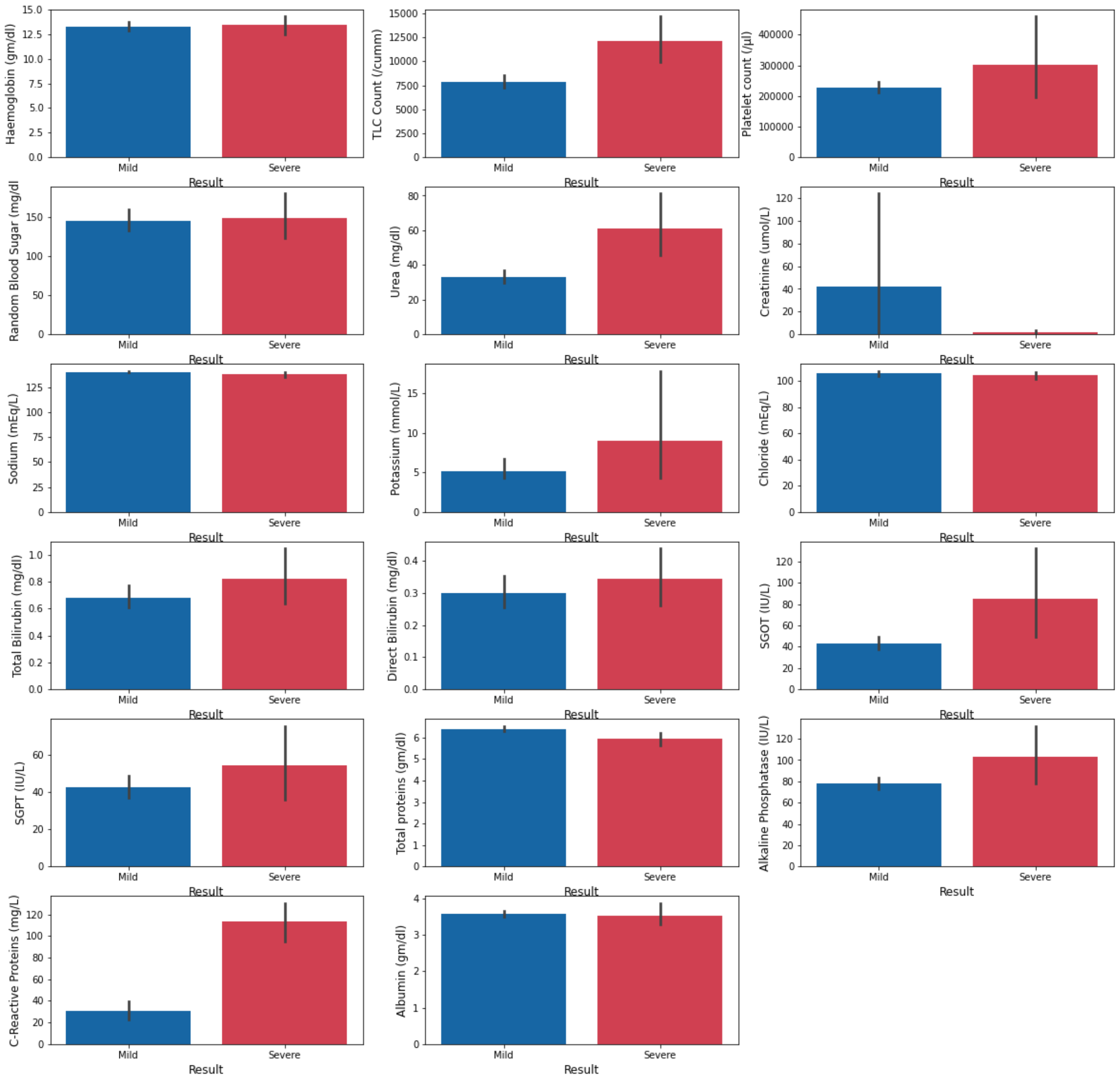

Supplementary Figure 8: Depicting the difference in the blood parameter levels of **mild and severe COVID-19 patients.** Black line showing standard deviation of bar plot.

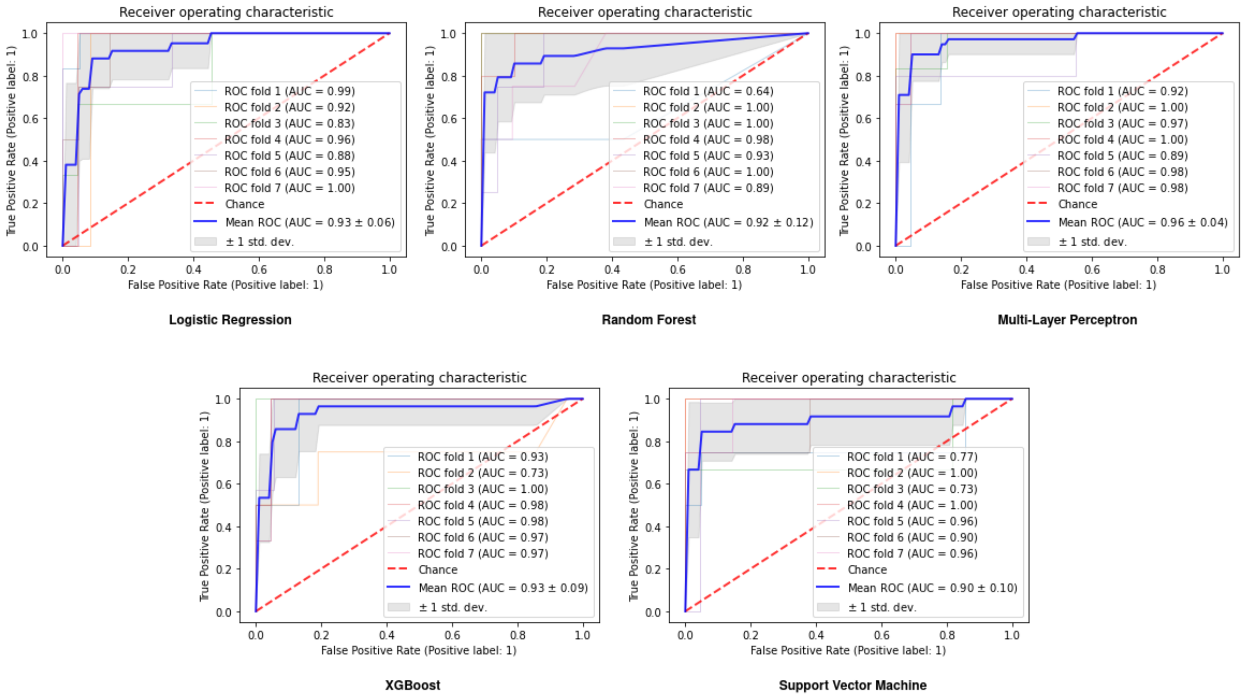

Supplementary Figure 9: ROC curves of all five machine learning models used in the study.
